# *Arabidopsis* RBV is a conserved WD40 repeat protein that promotes microRNA biogenesis and ARGONAUTE1 loading

**DOI:** 10.1038/s41467-022-28872-x

**Published:** 2022-03-08

**Authors:** Chao Liang, Qiang Cai, Fei Wang, Shaofang Li, Chenjiang You, Chi Xu, Lei Gao, Dechang Cao, Ting Lan, Bailong Zhang, Beixin Mo, Xuemei Chen

**Affiliations:** 1 Guangdong Provincial Key Laboratory for Plant Epigenetics, College of Life Sciences and Oceanography, Shenzhen University, Shenzhen, 518060 China; 2 College of Horticulture, College of Life Sciences, Haixia Institute of Science and Technology, Fujian Agriculture and Forestry University, Fuzhou, 350002 China; 3 College of Biological Sciences, China Agricultural University, Beijing, 100193 China; 4 State Key Laboratory of Genetic Engineering and Collaborative Innovation Center of Genetics and Development, Institute of Plant Biology, School of Life Sciences, Fudan University, Shanghai, 200438 China; 5 Department of Botany and Plant Sciences, Institute for Integrative Genome Biology, University of California, Riverside, CA 92521 USA

**Keywords:** Plant molecular biology, Gene regulation, RNA metabolism

## Abstract

MicroRNAs (miRNAs) play crucial roles in gene expression regulation through RNA cleavage or translation repression. Here, we report the identification of an evolutionarily conserved WD40 domain protein as a player in miRNA biogenesis in *Arabidopsis thaliana*. A mutation in the *REDUCTION IN BLEACHED VEIN AREA* (*RBV*) gene encoding a WD40 domain protein led to the suppression of leaf bleaching caused by an artificial miRNA; the mutation also led to a global reduction in the accumulation of endogenous miRNAs. The nuclear protein RBV promotes the transcription of *MIR* genes into pri-miRNAs by enhancing the occupancy of RNA polymerase II (Pol II) at *MIR* gene promoters. RBV also promotes the loading of miRNAs into AGO1. In addition, RNA-seq revealed a global splicing defect in the mutant. Thus, this evolutionarily conserved, nuclear WD40 domain protein acts in miRNA biogenesis and RNA splicing.

## Abstract (web summary)

MicroRNAs regulate gene expression through RNA cleavage or translation repression. Here the authors show that RBV, an evolutionarily conserved WD40 domain protein, acts to promote *MIR* transcription, pri-miRNA processing and miRNA loading into AGO1.

## Introduction

MicroRNAs (miRNAs), 20–24 nucleotides (nt) in length, are one class of endogenous noncoding small RNAs in eukaryotes. miRNAs are players in gene regulatory networks involved in many biological processes such as development, metabolism, and immunity in plants^1^. Target gene expression is regulated by miRNAs post-transcriptionally through RNA cleavage or translation repression^2^.

The biogenesis of plant miRNAs entails a series of steps. Genes encoding miRNAs (*MIR*) are transcribed into pri-miRNAs that form imperfect stem–loop structures by DNA-dependent RNA polymerase II (Pol II)^3,4^. *MIR* transcription is facilitated by Mediator^5^, (NOT2)^6^, CELL DIVISION CYCLE5 (CDC5)^7^, Elongator^8^, the TREX-2 component THP1^9^, and the chromatin remodeling factor CHR2^10^. Pri-miRNAs are processed into pre-miRNAs by DICER-LIKE1 (DCL1)^1,11–13^. In humans, pri-miRNAs are thought to be co-transcriptionally processed, as they are associated with chromatin during transcription and processed at sites of transcription before splicing^14,15^. It is proposed that the retention of pri-miRNAs at transcription sites may enhance processing^15^. In *Arabidopsis*, NOT2, CDC5, and Elongator interact with both Pol II and DCL1^6–8^, suggesting that pri-miRNA transcription and processing may be also coordinated in plants. CHR2 probably also acts co-transcriptionally to repress the processing of pri-miRNAs^10^.

The efficient processing of pri-miRNAs requires the double-stranded RNA-binding protein HYPONASTIC LEAVES1 (HYL1)^16,17^ and the zinc finger protein SERRATE (SE)^18,19^, which form the microprocessor complex with DCL1^20,21^. The three proteins as well as pri-miRNAs are also found in nuclear foci called dicing-bodies (D-bodies)^22^. Pre-miRNAs are processed to miRNA/miRNA* duplexes, which undergo 3′ methylation by the methyltransferase HEN1 to maintain miRNA stability^23^. Finally, the mature miRNA strands associate with ARGONAUTE1 (AGO1) to form RNA-induced silencing complexes (RISCs), which are active forms of miRNAs^24–26^. In Arabidopsis, size exclusion chromatography showed that the molecular weight of miRISCs is similar to that of AGO1, suggesting that miRISCs are bi-molecular AGO1-miRNA complexes, although larger complexes containing AGO1 can also be detected^24,27,28^.

Here, we isolated a mutant with global defects in miRNA biogenesis in *Arabidopsis*. The mutation is in a previously uncharacterized gene encoding a protein containing seven WD40 repeats, which we designate as *RBV*. *RBV* promotes the transcription of *MIR* genes, as loss of function of *RBV* reduces *MIR* promoter activity and the occupancy of Pol II at *MIR* promoters. In addition, *RBV* promotes the localization of HYL1 in D-bodies. On the basis of these results, we propose that RBV may act to coordinate *MIR* transcription and pri-miRNA processing in plant miRNA biogenesis. Moreover, the association of miRNAs with AGO1 was drastically decreased and AGO1 resided in complexes larger than miRISCs in the *rbv-1* mutant, suggesting that RBV promotes the loading of miRNAs into AGO1. *RBV* also has a global role in pre-mRNA splicing, affecting a set of short introns.

## Results

### Isolation of a mutant with defects in miRNA biogenesis

**Fig. 1:**
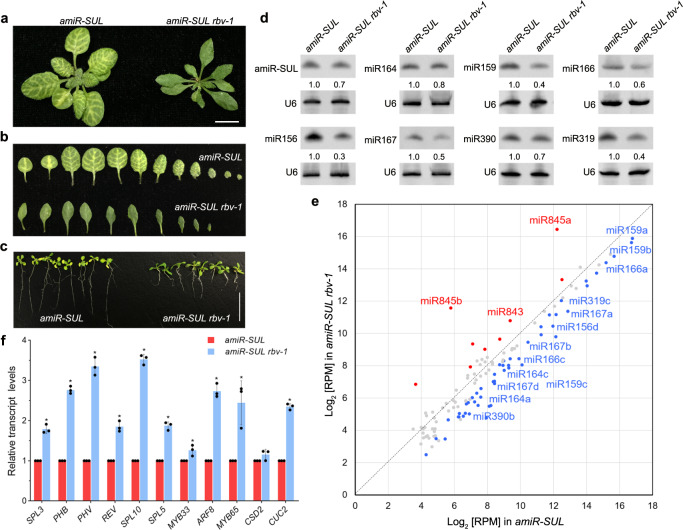
Isolation of the silencing suppressor mutant *rbv-1* from an *amiR-SUL* line. **a** Phenotypes of 1-month-old *amiR-SUL rbv-1* and *amiR-SUL* plants. Bar = 1 cm. **b** Images of rosette leaves from 1-month-old plants grown under long-day conditions. **c** 14-day-old seedlings showing reduced root length in *amiR-SUL rbv-1*. Bar = 1 cm. **d** RNA gel analysis showing reduced accumulation of amiR-SUL and endogenous miRNAs in the *amiR-SUL rbv-1* mutant. U6 was used as an internal control. The numbers represent relative abundance. Two independent repeats gave similar results. **e** A scatter plot showing the abundance of miRNAs in *amiR-SUL rbv-1* and *amiR-SUL* as determined by small RNA-seq with 14-day-old seedlings. miRNA abundance was calculated as reads per million mapped reads (RPM) and miRNAs with RPM > 10 in either genotype are shown. The red dots indicate miRNAs with higher levels in *amiR-SUL rbv-1*, and the blue dots indicate miRNAs with lower levels in *amiR-SUL rbv-1*. (Student’s *t* test, *P < 0.05). **f** Determination of miRNA target mRNA levels in *amiR-SUL* and *amiR-SUL rbv-1* in 14-day-old seedlings by RT-qPCR. *UBQUITIN5* (*UBQ5*) was used as the internal control. The values were relative to those in *amiR-SUL*. Error bars represent standard deviation from three technical replicates. Asterisks indicate a significant difference between *amiR-SUL* and *amiR-SUL rbv-1* (Student’s *t* test, *P < 0.05). Source data are provided as a Source Data file.

We performed an ethylmethane sulfonate mutagenesis screen for *Arabidopsis* mutants in miRNA biogenesis, utilizing the vein-centered leaf beaching phenotype caused by the phloem-specific expression of an artificial miRNA (amiR-SUL) targeting the * SULFUR* (*SUL*) gene as a visible marker for miRNA activity^29^. Several mutants with reduced leaf bleaching were isolated and found to be in genes with known roles in miRNA biogenesis/activity, such as *hyl1–11*, *dcl1–30*, *hen1–11*, and *ago1–25*, suggesting that the genetic screen was effective (Supplementary Fig. 1a, b). The *ago1–25* allele isolated in our study was thus named because it harbored the same mutation  as the one previously reported^30^. In addition, we isolated a new suppressor mutant with reduced leaf bleaching (Fig. 1a, b); the mutation was designated *rbv-1* as it was later shown to be in a previously uncharacterized gene that we named *REDUCTION IN BLEACHED VEIN AREA* (*RBV*). This mutant exhibited pleiotropic developmental phenotypes, such as reduced root length, smaller plant size, narrow leaves, short stature, increased branching, and reduced fertility (Fig. 1c and Supplementary Fig. 2). Northern blot analyses showed that both amiR-SUL and endogenous miRNAs (miR156, miR159, miR164, miR165, miR167, miR319, and miR390) were moderately reduced in abundance in 14-day-old *amiR-SUL rbv-1* seedlings as compared to *amiR-SUL* seedings (Fig. 1d). We also performed small RNA sequencing with 14-day-old *amiR-SUL* and *amiR-SUL rbv-1* seedlings. Clustering analysis showed that the three biological replicates for each genotype were highly reproducible (Supplementary Fig. 3a). Reads corresponding to miRNAs were normalized against total mapped reads and miRNAs with RPM (reads per million mapped reads) > 10 in either genotype (average of three replicates) were included in our analyses (Supplementary Data 1). An overall reduction in miRNA abundance in the mutant relative to the *amiR-SUL* line was observed (Fig. 1e and Supplementary Fig. 3b), while there were some miRNAs such as miR845a and miR845b showing increased accumulation in the *amiRSUL rbv-1* mutant. We also performed RT-qPCR to examine the expression of some miRNAs’ target genes. The transcript levels of *SPL3* and *SPL10* (targets of miR156), *PHB*, *REV*, and *PHV* (targets of miR165/6), *MYB33* and *MYB65* (targets of miR159), *ARF8* (a target of miR167), and *CUC2* (a target of miR164) were de-repressed in the *amiR-SUL rbv-1* mutant (Fig. 1f).

### *RBV* encodes an evolutionarily conserved WD40 domain protein

**Fig. 2:**
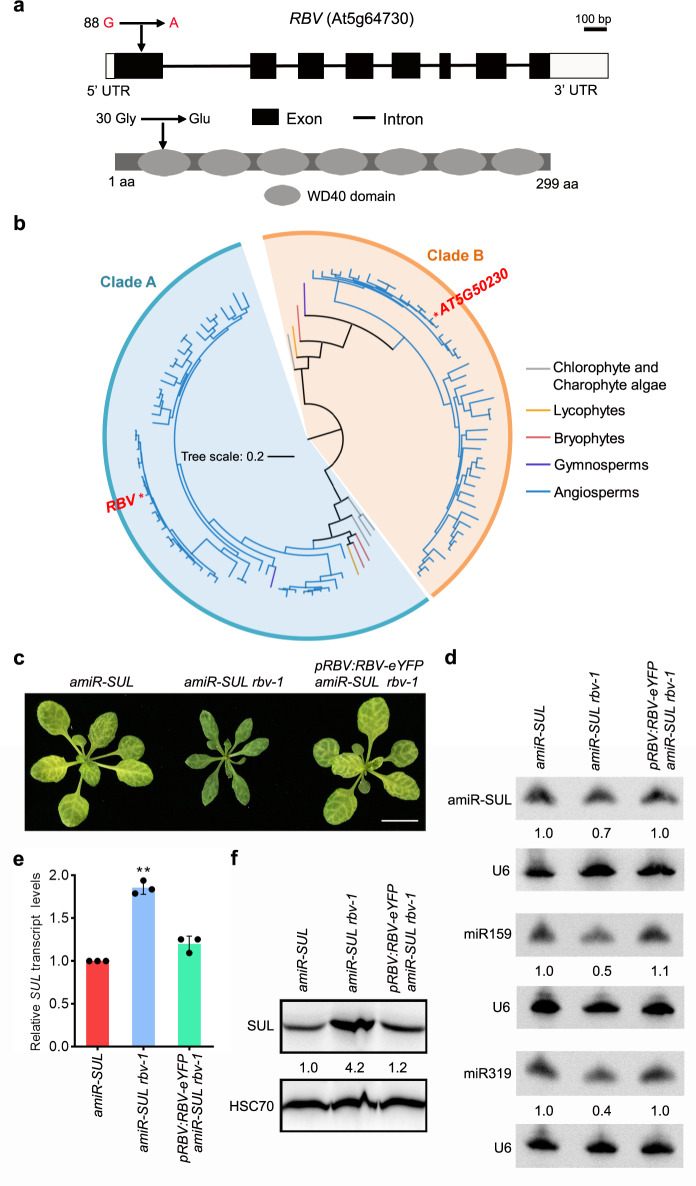
Identification of AT5G64730 as *RBV*. **a** Diagrams of the *RBV* (AT5G64730) gene (upper panel) and protein (lower panel). Rectangles and lines represent exons and introns, respectively. Black and white rectangles represent the coding region and the UTRs, respectively. The point mutation in *rbv-1* and the corresponding change at the amino acid level are indicated (arrows). The protein domains were predicted (http://smart.embl-heidelberg.de/). **b** A phylogenetic tree of *RBV* and its paralog *At5g50230* in plants. The colors of the branches represent different lineages of plant species. All genes used in the analysis are listed in Supplementary Data 2. The detailed phylogenetic tree is shown in Supplementary Fig. 4a. **c** Three-week-old plants of the indicated genotypes. *pRBV:RBV-eYFP* was introduced into *amiR-SUL rbv-1*. Bar = 1 cm. **d** RNA gel blot analysis of miRNAs from *amiR-SUL*, *amiR-SUL rbv-1*, and the complementation line *pRBV:RBV-eYFP amiR-SUL rbv-1* using 14-day-old seedlings. U6 was used as an internal control. The numbers represent relative abundance. **e** RT-qPCR to determine RNA levels of the amiR-SUL target gene *SUL* in the indicated genotypes. Three independent biological replicates were used for the calculation of standard deviation. (two-tailed Student’s *t* test, **P < 0.01). **f** Protein gel blot analysis to determine the protein levels of the amiR-SUL target gene *SUL* in the indicated genotypes. Two independent repeats gave similar results. Source data are provided as a Source Data file.

The *amiR-SUL rbv-1* mutant was backcrossed with the parental *amiR-SUL* line. In a total of 614 F2 plants, 141 (23%) exhibited the mutant phenotypes, which is consistent with the phenotype being caused by a single, nuclear, and recessive mutation (*χ*^2^ = 1.357; *P* = 0.244; (Supplementary Table 1)). In order to identify the causal mutation in *amiR-SUL rbv-1*, pooled DNA from mutant plants in the F2 of the *amiR-SUL rbv-1* x *amiR-SUL* cross was used for whole-genome re-sequencing. The results revealed that the *amiR-SUL rbv-1* phenotype was linked to a single nucleotide change (G-to-A) in the first exon of AT5G64730, causing the change of the encoded amino acid from glycine to glutamic acid (see Methods; Fig. 2a).

In order to confirm that *RBV* is indeed AT5G64730, a construct of *RBV* (AT5G64730)-*eYFP* driven by its native promoter was generated and introduced into *amiR-SUL rbv-1*. Phenotypes of the transgenic plants showed that *pRBV:RBV-eYFP* fully rescued the morphological defects of *amiR-SUL rbv-1* (Fig. 2c). Furthermore, the transgene restored amiR-SUL accumulation as well as leaf bleaching in *amiR-SUL rbv-1* (Fig. 2d). While the expression of the amiR-SUL target gene, *SUL*, was increased in the mutant at the RNA and protein levels, the transgene also restored *SUL* RNA (Fig. 2e) and SUL protein (Fig. 2f) in *amiR-SUL rbv-1* to wild-type levels. Moreover, the transgene rescued the defects in miR159 and miR319 accumulation in the *amiR-SUL rbv-1* mutant (Fig. 2d). Therefore, the miRNA biogenesis and morphological defects of *amiR-SUL rbv-1* were attributable to the mutation in *RBV* (AT5G64730).

*RBV* encodes a previously uncharacterized protein with seven WD40 repeats (Fig. 2a). In plants, WD40 repeat proteins are numerous, interact with diverse proteins, and act in a variety of biological processes, such as plant development and immunity^31,32^. To investigate whether RBV is an evolutionarily conserved protein, a phylogenetic tree in different plants was generated including Angiosperms, Gymnosperms, Bryophytes, Lycophytes, Chlorophyte, and Charophyte algae. In general, Arabidopsis *RBV* and its nearest paralog (*AT5G50230*) were separated in the latest common ancestor of multicellular algae (Fig. 2b). A detailed phylogenetic tree and the accession numbers of the proteins used can be found in Supplementary Data 2 and Supplementary Fig. 4a. According to the phylogenetic analysis, *RBV* orthologs can be found in plants ranging from single-cell green algae to core eudicots and grasses, and there is no close paralog of *RBV* in the latest common ancestor of land plants. In most eudicots that have undergone gamma whole-genome duplication (WGD) and Brassicaceae that have also undergone beta and alpha WGDs, *RBV* remained as a single copy. Only in species that have undergone recent, specific WGDs, such as apple, soybean, and maize, there are multiple copies of *RBV* (Supplementary Fig. 4a). This result indicated that *RBV* maybe functionally conserved and extra copies might be deleterious.

We also obtained two lines with T-DNA insertions in or near *RBV* (Salk_126634 and Salk_075672) (Supplementary Fig. 5a, c). Interestingly, the phenotypes of the T-DNA mutants were the same as wild type (Supplementary Fig. 5b). Next, we examined *RBV* transcripts in the two T-DNA insertion lines. RT-PCR showed that transcripts corresponding to the full-length coding region of *RBV* were present in the two mutants (Supplementary Fig. 5d). In addition, real-time RT-PCR showed that the levels of *RBV* transcripts were only mildly reduced in the two mutants (Supplementary Fig. 5e**)**, consistent with the lack of morphological phenotypes.

**Fig. 3:**
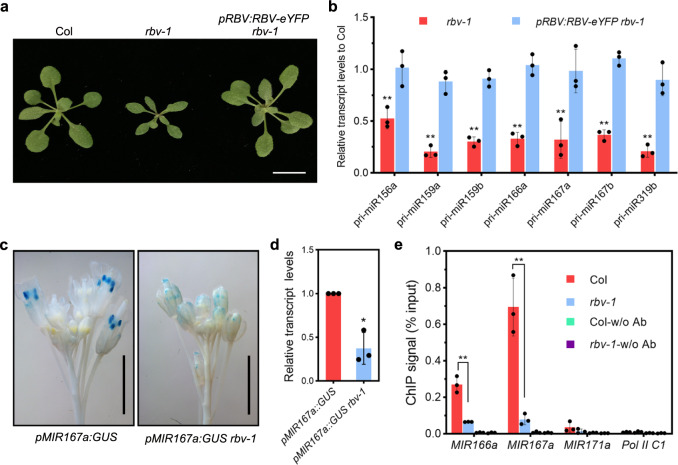
*RBV* promotes the transcription of *MIR* genes. **a** Three-week-old plants of the indicated genotypes. Bar = 1 cm. **b** Levels of seven pri-miRNAs in 14-day-old seedlings of *rbv-1* and the complementation line *pRBV:RBV-eYFP rbv-1* as determined by RT-qPCR. *UBQ5* was used as the internal control. Error bars represent standard deviation calculated from three independent replicates. (Student’s *t* test, **P < 0.01). **c** Representative images of GUS staining of *pMIR167a:GUS* and *pMIR167a:GUS rbv-1* inflorescences. Bars = 2 mm. **d** Transcript levels of GUS from the two genotypes as determined by RT-qPCR. The expression values were relative to *pMIR167a:GUS*. Error bars represent standard deviation calculated from three independent replicates. (two-tailed Student’s *t* test, *P < 0.05). **e** *RBV* is required for the recruitment of Pol II to *MIR166a* and *MIR167a* promoters. The occupancy of Pol II at various regions was determined by ChIP with *rbv-1* and Col using an antibody that recognizes the C-terminal repeat (YSPTSPS) of the largest subunit of Pol II. ChIP performed without the antibody served as a negative control. A genomic region between the genes AT2G17460 and AT2G17470 named Pol II C1 was also used as a negative control. Mean and standard deviation from three independent replicates are presented. (Student’s *t* test, **P < 0.01). Source data are provided as a Source Data file.

In order to remove the *amiR-SUL* transgene background, we crossed the *amiR-SUL rbv-1* mutant with wild-type (Col) plants. In the F2 population, we identified homozygous *rbv-1* plants without the *amiR-SUL* transgene through genotyping; these plants showed the same pleiotropic phenotypes as *amiR-SUL rbv-1* (Fig. 3a). The morphological phenotypes of *rbv-1* were completely rescued by the *pRBV:RBV-eYFP* transgene (Fig. 3a). We further confirmed that the *rbv-1* mutant without the *amiR-SUL* transgene had defects in miRNA accumulation. RNA gel blots showed that the levels of several endogenous miRNAs, such as miR156, miR159, miR164 and miR167, were reduced in abundance in *rbv-1* as compared to wild-type plants. Other miRNAs, such as miR319 and miR398, were unaffected (Supplementary Fig. 6a). To rule out the possibility that RBV indirectly regulated miRNA accumulation by affecting the expression of the miRNA biogenesis machinery, we examined the expression of the known genes involved in miRNA biogenesis, and no significant changes were observed in *rbv-1* (Supplementary Fig. 6b, c).

### *RBV* promotes the transcription of *MIR* genes

We next investigated how *RBV* promotes miRNA biogenesis. *MIR* gene transcription to produce pri-miRNAs is the first step in miRNA biogenesis. We first sought to determine pri-miRNA levels by RNA-seq in Col and *rbv-1*, each with three replicates. Of the 298 pri-miRNAs annotated in Araport11, only 41 gave reads in any sample. Pri-miRNAs with RPKM > 10 in either *rbv-1* or Col was used for differential expression analysis. Six pri-miRNAs were significantly altered in *rbv-1* relative to Col with four being decreased and two increased in abundance with *P* < 0.05 and fold-change > 1.5 as the cutoff (Supplementary Fig. 7). As many pri-miRNAs appeared to be at lower levels in the *rbv-1* mutant although they did not pass the cutoff (Supplementary Fig. 7), we performed RT-qPCR to determine the levels of seven pri-miRNAs in Col, *rbv-1* and *pRBV:RBV-eYFP rbv-1*. The levels of these pri-miRNAs were reduced to ~30-50% of wild-type levels in the *rbv-1* mutant and the reduction was rescued by *pRBV:RBV-eYFP* (Fig. 3b). The reduction in pri-miRNA abundance could be attributed to impaired *MIR* genes transcription, reduced stability of pri-miRNAs or enhanced pri-miRNA processing. To determine whether transcription was affected in the *rbv-1* mutant, we crossed *rbv-1* with a GUS reporter line (*pMIR167a:GUS*) under the control of the *MIR167a* promoter and obtained *pMIR167a:GUS rbv-1* (with both the transgene and the mutation being homozygous). GUS activity was visibly lower in *pMIR167a:GUS rbv-1* than in *pMIR167a:GUS* as revealed by GUS staining (Fig. 3c). RT-qPCR analysis confirmed that the *rbv-1* mutant had lower GUS transcript levels (Fig. 3d). Thus, reduced transcription of *MIR* genes could be one of the problems in miRNA biogenesis in *rbv-1*.

To further confirm a positive role of *RBV* in *MIR* gene transcription, the occupancy of Pol II at *MIR* loci was determined by chromatin immunoprecipitation (ChIP) with an antibody against the Pol II C-terminal repeats. ChIP without an antibody served as the negative control. *MIR166a*, *MIR167a*, and *MIR171a* promoter regions, as well as C1 (a region between the genes AT2G17460 and AT2G17470 known to not engage Pol II^7^), were examined by RT-qPCR after ChIP. The *MIR166a* and *MIR167a* promoter regions were enriched in the immunoprecipitates in both *rbv-1* and Col relative to C1 (Fig. 3e). Pol II occupancy at *MIR166a* and *MIR167a* promoter regions was reduced in the *rbv-1* mutant relative to Col, while the signal on *MIR171a* was too low to be calculated (Fig. 3e). Thus, *RBV* facilitates the recruitment of Pol II to *MIR* loci.

### RBV is localized in the nucleoplasm and required for the proper localization of HYL1 in D-bodies

**Fig. 4:**
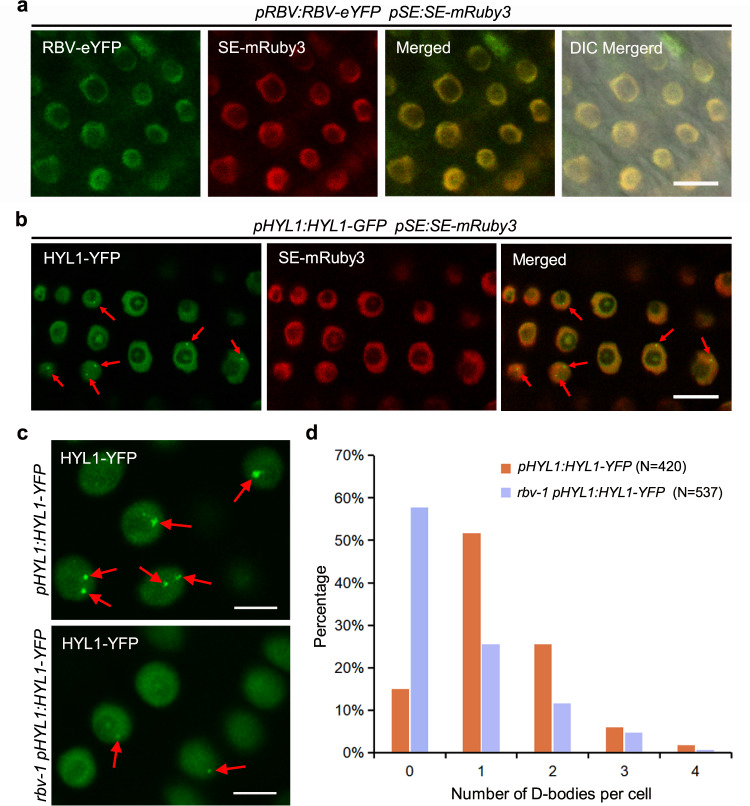
RBV is required for the proper localization of HYL1 in D-bodies. **a** RBV is localized in the nucleoplasm. eYFP and mRuby3 signals were detected in root cells (*n* = 100) from *pRBV:RBV-eYFP pSE:SE-mRuby3* transgenic plants. Bar = 10 μm. **b** HYL1 and SE protein localization in roots of *pHYL1:HYL1-YFP pSE:SE-mRuby3* plants. Both proteins show nucleoplasmic localization while HYL1 also shows D-body localization. In total 100 cells were observed. Bar = 10 μm. **c** Representative images of *pHYL1:HYL1-YFP* signals in root cells from the meristematic zone in the two genotypes. Arrows indicate D-bodies. Bars = 5 μm. **d** The percentage of cells with 1–4 D-bodies per cell in wild type and *rbv-1*. The *x*-axis represents the number of D-bodies per cell, and the *y*-axis represents the percentage of cells with the corresponding number of D-bodies. “*N*” means the numbers of total root cells examined. Source data are provided as a Source Data file.

We studied the expression of *RBV* in various tissues and the subcellular localization of the protein. RT-PCR analyses of RNAs from seedlings, roots, cauline leaves, rosette leaves, stems, and inflorescences showed ubiquitous *RBV* expression (Supplementary Fig. 8a). *RBV:RBV-eYFP* transgenic plants exhibited YFP fluorescence in the nucleoplasm but not the nucleolus (Fig. 4a). SE is a component of the microprocessor in pri-miRNA processing and a mRuby3-tagged SE protein driven by the *SE* promoter was also localized in the nucleoplasm, as previously reported^9^ (Fig. 4a). The nucleoplasmic localization of the two proteins prompted us to ask whether RBV interacts with SE. However, results of yeast two-hybrid, BiFC, and co-IP experiments did not show interactions between the two proteins (Supplementary Fig. 8c–e).

HYL1 and DCL1 form nuclear foci known as D-bodies, which are sites of pri-miRNA processing^22,33^. To determine the status of D-bodies in the *rbv-1* mutant, we crossed a *pHYL1:HYL1-YFP* transgene^22^ into the mutant and obtained plants homozygous for both the transgene and the *rbv-1* mutation. D-body numbers were determined in 420 and 537 root nuclei of wild-type and *rbv-1* plants, respectively. The number of HYL1-YFP D-bodies was significantly decreased in *rbv-1* (Fig. 4c, d), suggesting that mutation of RBV leads to a defect in D-body formation or the localization of HYL1 to D-bodies. Although SE was reported to form D-bodies in *N. benthamiana*^22^ and Arabidopsis^33^, we have never observed SE D-bodies or nuclear speckles in Arabidopsis roots, no matter when SE was fused to mRuby or eCFP (Fig. 4a and Supplementary Fig. 8b). Even upon co-expression of *pSE:SE-mRuby3* and *pHYL1:HYL1-YFP*, SE D-bodies cannot be observed despite obvious HYL1 D-bodies (Fig. 4b).

### Mutation of *RBV* leads to a defect of miRNA loading into AGO1

**Fig. 5:**
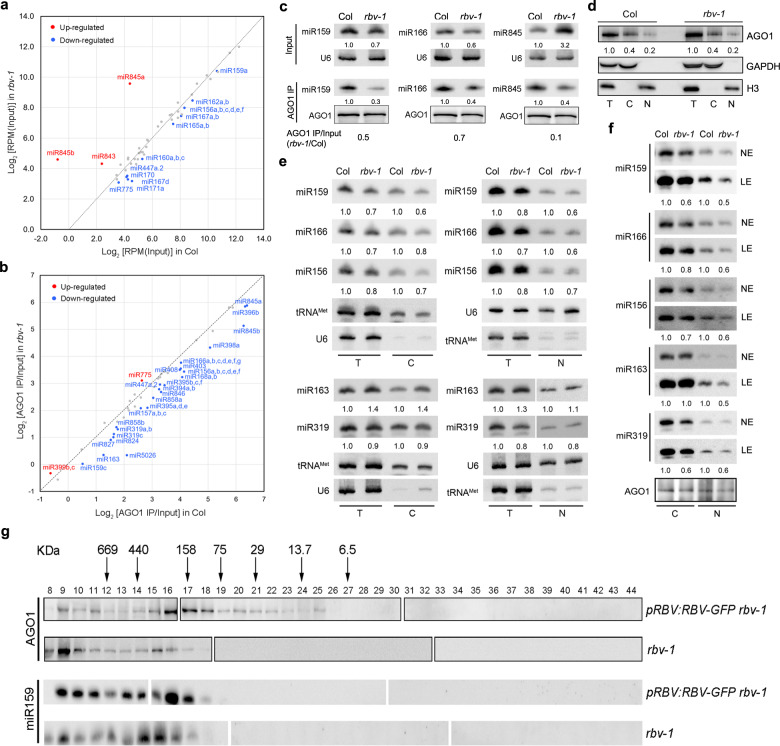
Mutation of *RBV* leads to a defect of miRNA loading into AGO1. **a** A scatter plot of miRNA abundance in *rbv-1* input vs. Col input. All miRNAs were normalized by total reads, and those with RPM > 10 in either genotype are shown. The red dots indicate miRNAs showing increased abundance in *rbv-1*, and the blue dots indicate miRNAs with reduced abundance in *rbv-1* (Student’s *t* test, *P < 0.05). **b** A scatter plot showing the AGO1 loading efficiency of miRNAs in *rbv-1* vs. Col as determined by AGO1 IP small RNA-seq. AGO1 loading efficiency is represented by the ratio of miRNA abundance in AGO1 IP vs. input. All miRNAs with RPM value > 10 in either genotype in the input samples (as in **a**) are shown here. The red dots indicate miRNAs with increased AGO1 association in *rbv-1*, and the blue dots indicate miRNAs with reduced AGO1 association in *rbv-1* (Student’s *t* test, *P < 0.05). **c** RNA gel blots analysis of three miRNAs before (input) and after AGO1 IP. U6 was used as an internal control for the input samples. For the IP samples, a portion was used for protein gel blot analysis to quantify AGO1 protein levels. The levels of miRNAs in the IP samples were normalized against AGO1 protein levels. No matter whether the assayed miRNAs were increased or reduced in abundance in input samples, they all showed reduced AGO1 association. Three independent repeats gave similar results. **d** Western blots to determine the nucleocytoplasmic partitioning of AGO1 in Col and *rbv-1*. T total extract, C cytoplasmic fraction, N nuclear fraction. Blots were analyzed using AGO1, GAPDH, and H3 antibodies, respectively. H3 was used as a nuclear marker in the quantification of AGO1 in the T and N samples. GAPDH was used as a cytoplasmic marker in the quantification of AGO1 in the T and C samples. Three independent repeats gave similar results. **e** Small-RNA gel blot analysis to determine th**e** levels of miRNAs from total extract (T) and from the cytoplasmic (C) and nuclear (N) fractions in Col and *rbv-1*. U6 and tRNA^Met^ served as nuclear and cytoplasmic RNA markers, respectively. They also served as the loading controls for the nuclear and cytoplasmic fractions for the quantification of miRNA levels. Two independent repeats gave similar results. **f** Small RNA gel blot analysis of miRNAs in AGO1 IP from the cytoplasmic (C) and nuclear (N) fractions. NE, normal exposure; LE long exposure. Two independent repeats gave similar results. **g** Size exclusion chromatography with *pRBV:RBV-eYFP rbv-1* and *rbv-1* samples followed by western blotting to detect AGO1 and northern blotting to detect miR159. The upper panel indicates the distribution of AGO1 while the lower panel represents the distribution of miR159 among the fractions. The numbers above the AGO1 blots indicate those of the fractions. Note that no AGO1 or miR159 was detected in fractions 1–7 (not shown). The positions of the molecular weight standards are shown above the AGO1 blots. Two independent repeats gave similar results. Source data are provided as a Source Data file.

miRNAs are loaded into AGO1 to form miRISCs that execute the silencing of target RNAs^24,26,34^. To determine whether *RBV* affects the formation of miRISCs, we performed AGO1 IP followed by sRNA-seq with both input and IP samples from wild-type and *rbv-1* seedlings. Three replicates were performed and showed high reproducibility (Supplementary Fig. 9). From input samples, many miRNAs showed a small but statistically significant reduction in abundance in the *rbv-1* mutant (Supplementary Data 3). A few miRNAs, such as miR163, miR845a, miR845b and miR843, were elevated in abundance in the mutant (Fig. 5a; Supplementary Data 3). The loading status of each miRNA was expressed as the ratio of miRNA abundance between AGO1 IP and input and differences between wild type and *rbv-1* were evaluated by student’s *t*-test. A global reduction in the AGO1 loading of miRNAs was observed in the *rbv-1* mutant (Fig. 5b; Supplementary Data 4). Notably, miR845a and miR845b, which exhibited elevated levels, were less associated with AGO1 in the mutant, indicating that RBV is crucial for miRISC formation. RNA gel blots were also performed to validate the sRNA-seq results. miR159 and miR166 levels were lower in input and further reduced in AGO1 IP in the mutant (Fig. 5c). miR845a levels were strongly increased in the *rbv-1* input sample as compared with Col input but greatly reduced in the *rbv-1* AGO1 IP sample as compared with Col AGO1 IP (Fig. 5c; Supplementary Data 4). Thus, the *rbv-1* mutant exhibits a global miRNA loading defect.

AGO1 is a nuclear-cytoplasmic shuttling protein and is thought to load miRNAs in the nucleus^25^. We performed nuclear-cytoplasmic fractionation to determine whether RBV affects the nuclear–cytoplasmic partitioning of AGO1. The results showed that the cytoplasmic/nuclear (C/N) ratios of AGO1 were similar between Col and the *rbv-1* mutant (Fig. 5d). We also detected miRNAs by gel blots in cytoplasmic and nuclear fractions. The results showed that miR159, miR166, miR156, and miR319 were similarly decreased in the nuclear and cytoplasmic fractions in *rbv-1* (Fig. 5e). Thus, RBV did not affect the nuclear–cytoplasmic partitioning of AGO1 or miRNAs. Next, we performed AGO1 IP followed by northern blotting to determine the miRNA loading efficiency. Using the ratio of miRNA abundance in AGO1 IP vs. input to represent miRNA loading efficiency, the calculated loading efficiencies for miR159, miR166, miR156, and miR319 were similarly reduced in the nuclear and cytoplasmic fractions in the *rbv-1* mutant, suggesting that this experiment at the steady-state level could not pinpoint the subcellular location of the AGO1 loading defect of the mutant. Intriguingly, for miR163 that showed increased abundance in both the nuclear and the cytoplasmic fractions in the mutant (Fig. 5e), the loading efficiencies were reduced in the nuclear but not the cytoplasmic fraction in *rbv-1* (Fig. 5f), which is indicative of a nuclear loading defect.

To determine how RBV might promote the loading of miRNAs into AGO1, we first examined whether RBV interacts with AGO1. Co-IP was performed with *pRBV:RBV-eYFP* plants using anti-GFP and anti-AGO1 antibodies, but no interaction between RBV and AGO1 was detected. We next sought to determine whether the bi-molecular feature of miRISCs is affected in the mutant. Protein extracts from *pRBV:RBV-eYFP rbv-1* and *rbv-1* plants were subjected to gel filtration followed by western blotting to detect AGO1 and northern blotting to detect miR159. Among the 44 fractions, AGO1 was distributed in both high molecular weight (HMW) (fractions 9–11) and low molecular weight (LMW) (fractions 16–17) complexes in *pRBV:RBV-eYFP rbv-1*, consistent with previous findings^28^. Notably, the LMW complexes, which corresponded to monomeric AGO1 in size, also showed peak levels of miR159, suggesting that they represent miRISCs with one AGO1 protein and one miRNA. However, such miRISCs were greatly reduced in *rbv-1*, with AGO1 being only in HMW complexes instead (Fig. 5g). The distribution of miR159 also shifted towards HMW complexes in *rbv-1* (Fig. 5g). Western blot analyses with anti-GFP antibody showed that RBV-eYFP was present in fractions 8–16 with estimated molecular weights much higher than that of an RBV-eYFP monomer, suggesting that RBV itself also resides in protein complexes (Supplementary Fig. 10). Therefore, RBV promotes the formation of miRISCs that contain only AGO1 and miRNAs. The HMW AGO1 complexes may represent intermediates in RISC formation.

AGO1 is known to associate with trans-acting small interfering RNAs (ta-siRNAs) in addition to miRNAs^24,26^. Contrary to miRNAs, which are loaded into AGO1 in the nucleus, ta-siRNAs are loaded into AGO1 in the cytoplasm^25^. We investigated whether RBV affects the ta-siRNA-AGO1 association. We quantified 21-nt siRNAs that mapped to 100-bp windows that overlapped with *TAS1A*, *TAS1B*, *TAS1C*, and *TAS2* loci. The ta-siRNAs were not significantly altered in *rbv-1* input as compared to Col input in each 100-bp window (Supplementary Fig. 11a, c, e, g). We then analyzed their levels in AGO-IP vs. input and found that the loading of ta-siRNAs into AGO1 was largely unaffected. Only ta-siRNAs in window 3 of *TAS2B* and window 3 of *TAS1C* were affected (Supplementary Fig. 11b, d, f, h). The levels of miR173, the trigger of ta-siRNA biogenesis from *TAS1* and *TAS2* loci, were not significantly different between Col and the *rbv-1* mutant (Supplementary Fig. 11i). The fact that ta-siRNA loading was largely unaffected in the mutant is consistent with RBV being a nuclear protein.

### RBV is required for the splicing of short introns in certain pre-mRNAs

In order to determine whether *RBV* affects the expression of protein-coding genes, we performed RNA-seq with 14-day-old seedlings of Col and *rbv-1* in triplicates. The three biological replicates for each genotype were highly reproducible (Supplementary Fig. 12).

Differentially expressed genes (DEGs) were identified between Col and mutant samples with FPKM > 1, fold-change > 2, and FDR < 0.05 as the cutoff. In total, we identified 632 upregulated (hyper-DEGs) and 363 downregulated (hypo-DEGs) genes, respectively (Supplementary Data 5 and 6, Supplementary Fig. 13a). Gene Ontology (GO) analyses showed that both the hyper-DEGs and hypo-DEGs were enriched in genes with roles in responses to stimuli (Supplementary Fig. 13b, c). We also examined the global transcript levels of miRNA targets from the RNA-seq data. Although several examined miRNA targets were found to be derepressed in the mutant by RT-qPCR, a global trend of increased levels of miRNA target transcripts was not observed (Supplementary Fig. 13d, Supplementary Data 7). It is possible that the *rbv-1* mutation leads to a defect in the transcriptional regulation of these genes as well.

**Fig. 6:**
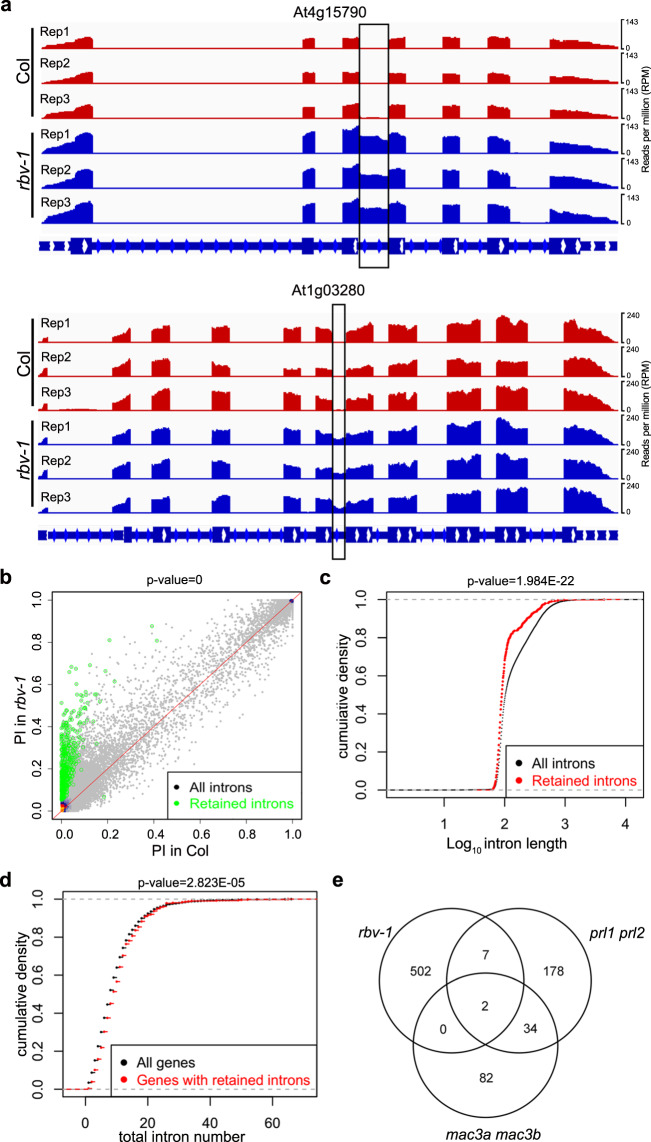
*RBV* function is required in the splicing of short introns in certain pre-mRNAs. **a** Examples of two genes with intron retention defects in the *rbv-1* mutant. RNA-seq reads are shown against the gene models below. In the gene models, rectangles and lines represent exons and introns, respectively. The black rectangles indicate retained introns in the *rbv-1* mutant. **b** A scatter plot showing percent retained introns (PI) in wild type and the *rbv-1* mutant. The green dots represent introns with statistically significant retention defects in the mutant (Wilcoxon test, *P* = 0). **c** Cumulative density plots of intron length for all introns and for retained introns in the *rbv-1* mutant (Wilcoxon test, *P* = 1.984E−22). **d** Cumulative density plots of intron number in all genes and for genes with retained introns in the *rbv-1* mutant. (Wilcoxon test, *P* = 2.823E−05). **e** Venn diagrams showing the numbers of retained introns in *rbv-1*, *prl1 prl2* and *mac3a mac3b* mutants, and the overlaps among the introns retained in these mutants.

It has been reported that MOS4-associated complex (MAC) components MAC3A, MAC3B, MAC7, and two WD40 repeat proteins (PRL1 and PRL2) affect both miRNA biogenesis and the splicing of protein-coding transcripts^35–37^. This prompted us to examine whether the *rbv-1* mutant had splicing defects using the RNA-seq data (see Methods). In total, 474 Genes were found to have intron retention defects in the *rbv-1* mutant compared to Col, with a total of 511 intron retention events (Supplementary Data 8). Two examples (At4g15790 and At1g03280) are presented in Fig. 6a. PI (percent of intron reads) was increased in the *rbv-1* mutant (Fig. 6b). No differential expression of the genes with intron retention was observed between the *rbv-1* mutant and wild type (Supplementary Fig. 14a). Next, we examined whether the genes with intron retention or the retained introns in the *rbv-1* mutant had any common features. Intriguingly, the retained introns in the *rbv-1* mutant seemed to be shorter as compared to the overall length distribution of introns (*P* = 1.984083e−22) (Fig. 6c). Besides, genes affected in *rbv-1* tended to have more introns than all genes (*P* = 2.823e−05) (Fig. 6d). Intron retention defects are also found in *prl1 prl2* and *mac3a mac3b* mutants^35^. We compared the retained introns in *rbv-1*, *prl1 prl2* and *mac3a mac3b* and found little overlap between the introns affected in *rbv-1* with those affected in either *prl1 prl2* or *mac3a mac3b* (Fig. 6e), suggesting that *RBV* affects different introns from the MAC components.

Many *MIR* genes have introns^38–40^. The RNA-seq did not detect intron retention events in pri-miRNAs, but the low abundance of pri-miRNAs could have precluded the detection of intron retention events. We performed RT-PCR to test the splicing of introns from three miRNA precursors (pri-miR163, pri-miR156, and pri-miR168) with intron-flanking primers. Genomic DNA was amplified with the same primers to indicate the size of the intron-containing fragments. No defects in pri-miRNA splicing were observed in the *rbv-1* mutant (Supplementary Fig. 14b).

## Discussion

RBV, a WD40 protein, is an evolutionarily conserved protein in plants. However, it has not been studied from any species. In this study, we show that a recessive mutation in *RBV* reduces the levels of many endogenous miRNAs, indicating that mutation of RBV leads to a defect in miRNA biogenesis. Consistent with the known roles of plant miRNAs in various developmental processes, the *rbv-1* mutant exhibits strong developmental defects. How does *RBV* promote miRNA biogenesis? Our findings suggest a role of *RBV* in promoting Pol II transcription of *MIR* genes, which is supported by the reduced levels of pri-miRNAs, compromised *MIR167a* promoter activity, and decreased Pol II occupancy at *MIR* genes in the *rbv-1* mutant. In Arabidopsis, a series of proteins have been found to promote *MIR* transcription and/or pri-miRNA processing and can be grouped into two main classes^1^. One group contains CBP80 and CBP20^41^, STA1^42^, SICKLE^39^, TOUGH^43^, PINP1^44^, THO1 and THO2^45,46^, and MOS2^47^. A common feature is that mutants in these genes show reduced levels of miRNAs and increased abundance of pri-miRNAs. The second group of proteins acts in a different manner. In loss-of-function mutants in the genes in this group, the abundance of both pri-miRNAs and mature miRNAs is reduced. Proteins in this group include DAWDLE^48^, CDC5^7^, NOT2^6^, Elongator^8^, PRL1^49^, MAC7^35^, PP4^37^, and THP1^9^. Besides, mutants in *PRL1*, *CDC5*, *MAC7*, *PP4*, and *THP1* show a reduced number of HYL1 D-bodies^7,9,35,37,49^. A number of proteins in this group interact with DCL1, HYL1, or SE and thus are thought to bridge *MIR* gene transcription and pri-miRNA processing^6–9,37,48^. Besides the two main classes, the third group, which includes CHR2, a partner of SE, promotes the transcription of *MIR* genes but represses miRNA accumulation by inhibiting pri-miRNA processing^10^. Our studies show that RBV belongs to the second group of proteins that promotes *MIR* gene transcription and possibly pri-miRNA processing.

**Fig. 7:**
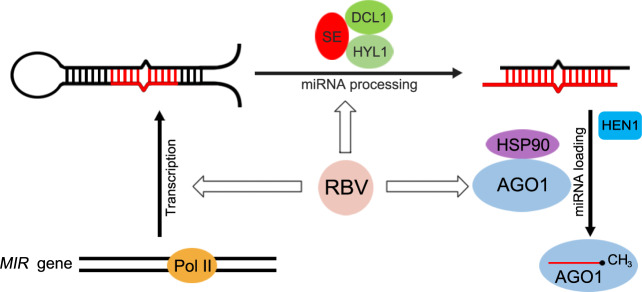
A model for the role of *RBV* in miRNA biogenesis. A pathway of miRNA biogenesis entailing *MIR* gene transcription, pri-miRNA processing, miRNA methylation, and miRISC formation is shown. Key protein players in each step are depicted as ovals. RBV promotes miRNA biogenesis at the *MIR* gene transcription and AGO1 loading steps and may also enhance pri-miRNA processing.

RBV also differs from the second group of proteins in that it has a clear role in miRISC formation. In the *rbv-1* mutant, both AGO1 and miR159 shift into complexes with higher molecular weights. RBV itself is also found in HMW complexes. We suspect that the HMW complexes containing AGO1 and miR159 represent intermediates in miRISC formation and that RBV helps the dissociation of AGO1-miRNA from other proteins to form active miRISCs. Heat Shock Protein 90 (HSP90) is required for sRNA loading into AGO1 in tobacco lysates^50^ and for RISC formation in Drosophila and humans^51^. HSP90 proteins are involved in RNA silencing in animals^52^ and plants^53,54^. Consistently, HSP90.4 was found in GFP-AGO1 IP spectrometry experiments^25^. Another protein that has recently been shown to play a role in miRISC formation is CARP9, which interacts with both HYL1 and AGO1 in the nucleus^55^. The relationship between RBV, HSP90 and CARP9 is worth investigating in the future. In summary, RBV, as a nuclear WD40 protein, is involved in multiple steps in miRNA biogenesis, including *MIR* transcription, pri-miRNA processing and AGO1 loading (Fig. 7).

Proteins such as MAC7, PP4, MAC3A/3B, and PRL1 act not only in miRNA biogenesis but also in pre-mRNA splicing in *Arabidopsis*^35,37^. We found that RBV also plays a role in splicing. Similar to mutants in *MAC7*, *PP4*, *MAC3A*/*3B*, and *PRL1*, no significant correlations between intron retention and changes in gene expression were found in the *rbv-1* mutant. Introns retained in the *rbv-1* mutant had minimal overlap with those in the other mutants, suggesting the presence of different categories of introns, whose splicing requires distinct factors. *RBV* acts on genes with more introns and introns that are shorter in length. Furthermore, intron retention was not found for intron-containing pri-miRNAs in *rbv-1*, suggesting that mutation of *RBV* leads to a defect in miRNA biogenesis and pre-mRNA splicing independently. It is possible that *RBV* function is required for yet other aspects of nuclear RNA metabolism.

## Methods

### Plant materials and growth conditions

The transgenic *Arabidopsis thaliana* (Col ecotype) line *pSUC2:amiR-SUL* expressing amiR-SUL under the *sucrose-proton symporter 2* (*SUC2*) promoter was a kind gift from Dr. Detlef Weigel^29^. Transgenic lines harboring *pSE:SE-mRuby3*, *pMIR167a:GUS*, and *pHYL1:HYL1-YFP*^5,9,22,49^ were used in this study. All seeds were sown on 1/2 Murashige and Skoog Basal Medium (Sigma-Aldrich, M5519) plus 1% Sucrose and 0.8% Agar. All plants were grown at 22 °C under 16 h light and 8 h dark cycles.

### Mutagenesis and mapping

EMS mutagenesis was performed as described^9^. A mutant, *amiR-SUL rbv-1*, with reduced leaf bleaching, was isolated and then backcrossed with the parental line *pSUC2:amiR-SUL*. In the F2 generation, ~100 plants with the *amiR-SUL rbv-1* phenotype were identified. Genomic DNA was extracted by the CTAB method^56^ from individual plants and an equal amount of DNA was pooled for genomic DNA library construction. The library was paired-end (PE151bp) sequenced on the Illumina platform HiSeq4000 at 50x coverage at BGI-Shenzhen, China. Focusing only on G-to-A mutations in coding regions, a mutation in At5g64730 was identified in *amiR-SUL rbv-1*. A derived cleaved amplified polymorphic sequences (dCAPS) marker was designed to genotype this mutation (Supplementary Table 2). The PCR products from wild type can be digested by *Nco*I, whereas those from *amiR-SUL rbv-1* could not. Genotyping the ~100 individuals showed that this mutation was linked to the plant phenotype.

### DNA constructs and complementation

The genomic region of *RBV* (At5g64730) including ~1 kb promoter was amplified with the primers proRBV-F and RBV-R (Supplementary Table 2) and cloned into the pTSK108 vector. The clone was sequenced to ensure the absence of mutations and was recombined with the pGWB640 gateway vector^57^ to generate the *pRBV:RBV-eYFP* construct via LR reaction. The *pRBV:RBV-eYFP* plasmid was transformed into the *rbv-1* mutant in both *amiR-SUL* and Col backgrounds through the *A. tumefaciens*-mediated floral dip method as described^58^. The T1 transgenic plants were selected by BASTA resistance.

### Fluorescence microscopy

For the visualization of RBV-eYFP and HYL1-YFP in transgenic plants, roots of 10-day-old seedlings were observed under a Zeiss LSM 5 Pascal inverted confocal microscope (excitation, 488/512 nm/nm; emission, 520/50 nm/nm). For SE-mRuby3 and SE-eCFP, roots of 10-day-old *pSE:SE-mRuby3* seedlings were observed under a LeicaSP8 confocal microscope (excitation, 514 nm; emission, 540–600 nm).

### Small RNA sequencing and data analysis

Total RNAs were extracted from 14-day-old seedlings using TRI reagent (TR118, Molecular Research Centre). Small RNAs in the size range of 15–40 nt were isolated from 30 μg total RNAs by resolving total RNAs in denaturing polyacrylamide gels, excising gel pieces containing 15–40 nt RNAs and elution of the small RNAs according to Liu et al^59^. Small RNA libraries were prepared with NEBNext Multiplex Small RNA Library Prep Set for Illumina (New England Biolabs, E7300), and then sequenced using an Illumina HiSeq2500 platform at BerryGenomics, China. Data analysis was performed with the pRNASeqTools pipeline (https://github.com/grubbybio/pRNASeqTools). The raw reads (SE50) were trimmed using Perl scripts to remove adapters (adapter: AGATCGGAAGAGC). The clean reads were mapped to the *A. thaliana* genome using the Bowtie program^60^. For miRNA analysis, the small RNA reads were mapped to miRBase v21. The sequences of miRNA and miRNA* were obtained from the PRIMEN database: http://www.pmiren.com/ftpdownload/Arabidopsis_thaliana_Ath/Arabidopsis_thalianamature.fa. For tasiRNA analysis, the reads that were mapped to 100-bp windows overlapping with *TAS1A*/*B*/*C* and *TAS2* loci were counted. The small RNA reads were normalized by calculating the RPM value (reads per million trimmed reads)^61^. The comparison between genotypes was conducted by the R package “DESeq2” with a 1.5-fold change and *P* < 0.01 as the cutoff^62^. For AGO1 IP sRNA-seq analysis, reads were normalized by total mapped reads. The comparison between genotypes was conducted with the two-factor model by DESeq2.

### Small RNA gel blotting

RNA gel analysis of small RNAs was performed as described^63^. Ten micrograms of total RNAs were separated on 15% polyacrylamide/8 M urea gels. After gel electrophoresis, the RNAs were transferred to a Hybond-NX nylon membrane (GE healthcare). Antisense complementary oligonucleotides (Supplementary Table 2) were synthesized with both 5’ and 3′ end-labeled biotin. A probe complementary to U6 (5′ CATCCTTGCGCAGGGGCCA 3′) was used to detect U6 as an internal control. Hybridization was performed for 16 h at 55 °C followed by washes. Signals were detected using the chemiluminescent nucleic acid detection module (Thermo Fisher, 89880).

### Reverse transcription-quantitative PCR (RT-qPCR)

RT-qPCR was performed to quantify mRNA and pri-miRNAs levels. One microgram total RNA was reverse transcribed with oligo (dT) using the PrimeScript^™^ 1st Strand cDNA Synthesis Kit (TAKARA, 6110A) according to the manufacturer’s instructions. RT-qPCR was performed in the 96-well StepOnePlus real-time system (ABI) using the SYBR premix ExTaq II kit (TAKARA, RR820A). The following scheme was used: 95 °C for 30 s and 40 cycles of 95 °C for 5 s and 60 °C for 30 s. The levels of transcripts were normalized to the level of the internal standard *UBQ5* and the 2^−△△^CT values of control samples were set to 1. The primers for pri-miRNAs are listed in Supplementary Table 2. Three independent biological replicates were carried out for each genotype. The student’s *t*-test was used for the evaluation of statistical significance.

### Histochemical GUS staining

Fourteen-day-old seedlings of *pMIR167a:GUS* and *pMIR167a:GUS rbv-1* (homozygous for both the transgene and the *rbv-1* mutation) were subjected to histochemical GUS staining according to the standard protocol^64^. Tissues were vacuum infiltrated in the staining solution (1 mM EDTA, 5 mM potassium ferricyanide, 5 mM potassium ferrocyanide, 100 mM sodium phosphate, 1% Triton X-100, 1 mg ml^−^^1^ X-Gluc) for 10 min and then incubated at 37 °C for 2 h in the dark. Tissue clearing was performed with 70% ethanol for 2 h before imaging.

### Chromatin immunoprecipitation (ChIP) assay

RNA Polymerase II ChIP was performed as described^5^ with 14-day-old Col and *rbv-1* seedlings using an antibody against RPB1 (Abcam, ab817, dilution 1:200). Seedlings were cross-linked in buffer (0.4 M Sucrose, 10 mM Tris-HCl Ph8.0, 1 mM EDTA, 1 mM PMSF, 1% proteinase inhibitor cocktail, and 1% formaldehyde) at room temperature under the vacuum and glycine was added to a final concentration of 100 mM to stop the crosslinking. Then the plant material was ground to a fine powder, which was then resuspended in cold nuclei-isolation buffer (0.25 M sucrose, 15 mM PIPES pH 6.8, 5 mM MgCl_2_, 60 mM KCl, 15 mM NaCl, 1 mM CaCl_2_, 0.9% Triton X-100, 1 mM PMSF and 1% proteinase inhibitor cocktail). The suspension was filtered through two layers of Miracloth and centrifuged at 12,000*g* for 20 min at 4 °C. The pelleted nuclei were resuspended in nuclei lysis buffer (50 mM HEPES pH7.5, 150 mM NaCl, 1 mM EDTA, 1% sodium dodecyl sulfate (SDS), 0.1% sodium deoxycholate, 1% Triton X-100, and 1% proteinase inhibitor cocktail) followed by sonication (Covaris S200). Then the sonicated suspension was centrifuged at 12,000*g* at 4 °C to pellet debris. The supernatant was collected and diluted with ChIP dilution buffer (50 mM HEPES pH7.5, 150 mM NaCl, 1 mM EDTA, 0.1% sodium deoxycholate, 1% Triton X-100, and 1% proteinase inhibitor cocktail). The diluted chromatin sample was precleared with protein A agarose beads followed by incubation with antibodies with gentle rotation overnight at 4 °C. Then protein A agarose beads were added followed by incubation for 2 h at 4 °C with gentle rotation. The beads were washed with low-salt wash buffer (150 mM NaCl, 20 mM Tris-HCl pH8.0, 0.2% SDS, 0.5% Triton X-100, and 2 mM EDTA), high-salt wash buffer (500 mM NaCl, 20 mM Tris-HCl pH8.0, 0.2% SDS, 0.5% Triton X-100 and 2 mM EDTA), and LiCl wash buffer (0.25 M LiCl, 1% sodium deoxycholate, 10 mM Tris-HCl pH8.0, 1% CA630 and 1 mM EDTA) and twice with TE buffer (1 mM EDTA, 10 mM Tris-HCl pH8.0). The chromatin was eluted with elution buffer (1 mM NaHCO_3_ and 1% SDS) and 20 µl 5 M NaCl was added followed by incubation at 65 °C for at least 6 h to reverse crosslinking. Removal of protein and RNA was achieved by adding 1.5 µl 18.9 mg ml^−1^ proteinase K and 1 µl of 1 M RNase A per 500 µl. DNA was extracted by the phenol/chloroform extraction method. RT-qPCR was performed with co-immunoprecipitated DNA, using primers listed in Supplementary Table 2. Relative enrichment was calculated by normalizing the amount of ChIP-ed DNA to the corresponding amount in the input.

### Western blot analysis

Western blots were performed as described^63^. Proteins from 14-day-old seedlings were extracted, resolved in 12% (v/v) SDS-polyacrylamide gels, and transferred to Hybond C-Extra membranes (Amersham Biosciences). The membranes were blocked with 5% (w/v) non-fat milk in Tris buffered saline tween (TBST) buffer and then probed with specific antibodies. Antibodies used included anti-GAPDH (Santa Cruz Biotechnology, sc-365062, dilution, 1:1000), anti-SUL (dilution, 1:1000)^35^, anti-HYL1 (Agrisera, AS06136, dilution, 1:2000), anti-AGO1 (Agrisera, AS09527, dilution, 1:2000), anti-SE (Agrisera, AS09532A, dilution, 1:1000), anti-H3 (Agrisera, AS10710, dilution 1:3000) and anti-GFP (Abcam, ab290, dilution, 1:3000). After three washes, the membranes were probed with horseradish peroxidase-conjugated, goat-anti-rabbit IgG (Bio-Rad, cat.172-1019) (dilution, 1:2000) or goat anti-mouse IgG (Bio-Rad, cat.170-6516, dilution, 1:2000). The protein signals were detected with the Amersham^TM^ ECL^TM^ Prime Western Blotting Detection Reagent (GE healthcare, RPN2232) and visualized with the Chemiluminescence imaging system (Clinx Science Instruments Co. Ltd., China).

### RNA-Seq data analysis

Total RNAs extracted from 14-day-old seedlings were sent for library construction at Novogene, China and libraries were sequenced on an Illumina Hiseq 4000 platform to generate paired-end reads of 150 bp in length. The data analysis of RNA-seq libraries was carried out as described^65^. The clean reads were collapsed into nonredundant ones and mapped to the Arabidopsis genome (ARAPORT11) using STAR, allowing a maximum of eight mismatches per paired-end read^66^. DEGs were identified between Col and *rbv-1* using cuffdiff^67^ with FPKM > 1, fold-change >2 and FDR < 0.05 as filters. Expression levels of all genes and DEGs were plotted using the value of log2 (FPKM + 1). GO enrichment analysis was performed with agriGO^68^ using a webtool (http://bioinfo.cau.edu.cn/agriGO/). Only the top 20 terms were presented in this paper. The analysis of splicing defects was carried out using Araport 11 intron annotation and a previously developed pipeline known as SQUID (https://github.com/sfli001/SQUID). In brief, the level of retained introns was calculated using two methods: PI_Junction (*intron*–*exon junction reads*/[*intron*–*exon junction reads* + *exon*–*exon reads*]) and PI_density (normalized intronic reads/normalized exonic reads). The differentially spliced introns were defined using a stringent cutoff: combined_FDR < 0.1, Diff_PI_Junction > 0.05, Diff_PI_Density > 0.05. PI_Junction was used to represent the levels of retained introns.

### AGO1 IP assay

One gram of 14-day-old seedlings was ground in liquid nitrogen, and IP buffer (50 mM Tris pH 7.5, 150 mM NaCl, 10% Glycerol, 0.1% NP-40, 1 mM PMSF) and EDTA-free protease inhibitor mixture (Roche) were added to the powder, which was followed by 20 min incubation with gentle shaking at 4 °C. The supernatant was incubated with 100 μL of dynabeads (Invitrogen, 10002D) for 2 h at 4 °C. After centrifugation, the supernatant was used for IP. 50 μL was saved as input, and the rest was incubated with anti-AGO1 antibody (Agrisera, AS09527, dilution, 1:2000) for 2 h at 4 °C. The beads were washed with wash buffer (IP buffer with 0.5% NP-40), and 1/10 (v/v) was added to 2× SDS-loading buffer for western blot analysis and 9/10 (v/v) was used for RNA isolation. Small RNA libraries were prepared using the NEBNext Multiplex Small RNA Library Prep Set for Illumina (New England Biolabs, E7300), and sequenced on an Illumina HiSeq2500 platform at BerryGenomics, China. The proteins were separated by SDS-PAGE and protein gel blot analysis was performed using anti-AGO1 antibody (Agrisera, AS09527, dilution, 1:2000).

### Nuclear–cytoplasmic fractionation

Twelve-day-old seedlings were collected and cross-linked in 0.5% formaldehyde/1× phosphate-buffered saline (PBS) buffer under vacuum for 10 min twice on ice. Then glycine was added to a final concentration of 100 mM to stop the crosslinking followed by incubation for 5 min under vacuum on ice. The plant material was washed in 1× PBS buffer and frozen in liquid nitrogen immediately for nuclear–cytoplasmic fractionation.^9^ The frozen seedlings were ground to a fine powder in liquid nitrogen and resuspended in 2 ml g^−1^ lysis buffer (20 mM Tris-HCl, pH7.5, 20 mM EDTA, 2.5 mM MgCl_2_, 25% glycerol, 250 mM sucrose, 5 mM DTT, and 1× protease inhibitor cocktail (Roche)). The suspension was filtered through two layers of Miracloth. The flow-through was centrifuged at 1500*g* for 10 min at 4 °C. The supernatant was centrifuged at 10,000*g* for 10 min at 4 °C, and the supernatant was collected as the cytoplasmic fraction, which was used for RNA isolation using the Trizol method. The pellet from the 1500*g* spin was washed 10 times with 10 ml nuclear resuspension buffer 1 (NRB1) (20 mM Tris-HCl, pH7.5, 2.5 mM MgCl_2_, and 0.2% Triton X-100). The washed pellet was resuspended with 500 µl NRB2 (20 mM Tris-HCl, pH7.5, 10 mM MgCl_2_, 250 mM sucrose, 0.5% Triton X-100, 5 mM β-mercaptoethanol, and 1× protease inhibitor cocktail). The sample was centrifuged at 16,000*g* for 45 min at 4 °C. The final nuclear pellet was used for RNA isolation using the Trizol method. The cytoplasmic and nuclear fractions underwent the same AGO1 IP procedure as described above.

### Gel filtration assay

One gram of 14-day-old T3 *pRBV: RBV-eYFP rbv-1* seedlings and wild type was collected and ground in liquid nitrogen. Then 1.5 ml phosphate buffer supplemented with 1 mM PMSF, 1% EDTA-free protease inhibitor mixture, RNase Inhibitor (TAKARA) and 0.4% CA630 were added to the powder. The homogenized crude extracts were kept on ice for 20 min. After two rounds of centrifugation for 20 min (12,000*g*) each at 4 °C, 1.5 ml of the cleared crude extract was immediately used for sample injection. Gel-filtration chromatography was carried out as described^28^ using a Superdex 200 Increase 10/300GL column. In total, 44 fractions (each 800 μL) were collected. A 300 μL of each fraction was used for protein extraction for detecting AGO1 and 500 μL for total RNA purification for sRNA detection. Markers of known sizes (Aprotinin, 6.5 kDa; Ribonuclease A, 13.7 kDa; Carbonic Anhydrase, 29 kDa; Conalbumin, 75 kDa; Aldolase, 158 kDa; Ferritin, 440 kDa; Thyroglobulin, 669 kDa; blue dextran, 2000 kDa) were used for column calibration.

### RBV conservation analysis

hmmsearch (HMMER 3.3) was employed to search for RBV homologs in a plant genome database (Supplementary Data 2) with a seed file created by the cDNA sequence of *RBV*. Close homologs of *RBV* were retained by a primary Neighbor-Joining tree based on MUSCLE (v3.8.1551)-aligned sequences and manual selection. The coding sequences of these genes were aligned by MUSCLE and manually corrected.

The final maximum likelihood tree was constructed using the general time-reversible model and 1000 bootstrap replicates based on nucleotide sequences. The output tree was visualized by iTOL^69^.

### Reporting summary

Further information on research design is available in the Nature Research Reporting Summary linked to this article.

## Supplementary information

Supplementary Information

Description of Additional Supplementary Files

Supplementary Data 1

Supplementary Data 2

Supplementary Data 3

Supplementary Data 4

Supplementary Data 5

Supplementary Data 6

Supplementary Data 7

Supplementary Data 8

Reporting Summary

## Data Availability

All raw and processed data were deposited at NCBI GEO (http://www.ncbi.nlm.nih.gov/geo/) with the accession number GSE152911. Source data are provided with this paper.

## Source data

Source Data

## References

[1] Yu Y, Jia T, Chen X (2017). The ‘how’ and ‘where’ of plant microRNAs. N. Phytol..

[2] Song X, Li Y, Cao X, Qi Y (2019). MicroRNAs and their regulatory roles in plant-environment interactions. Annu. Rev. Plant Biol..

[3] Xie Z (2005). Expression of Arabidopsis MIRNA genes. Plant Physiol..

[4] Zheng B (2009). Intergenic transcription by RNA polymerase II coordinates Pol IV and Pol V in siRNA-directed transcriptional gene silencing in Arabidopsis. Genes Dev..

[5] Kim YJ (2011). The role of mediator in small and long noncoding RNA production in *Arabidopsis thaliana*. EMBO J..

[6] Wang L (2013). NOT2 proteins promote polymerase II-dependent transcription and interact with multiple MicroRNA biogenesis factors in Arabidopsis. Plant Cell.

[7] Zhang S, Xie M, Ren G, Yu B (2013). CDC5, a DNA binding protein, positively regulates posttranscriptional processing and/or transcription of primary microRNA transcripts. Proc. Natl Acad. Sci. USA.

[8] Fang X, Cui Y, Li Y, Qi Y (2015). Transcription and processing of primary microRNAs are coupled by elongator complex in Arabidopsis. Nat. Plants.

[9] Zhang B (2020). Linking key steps of microRNA biogenesis by TREX-2 and the nuclear pore complex in Arabidopsis. Nat. Plants.

[10] Wang Z (2018). SWI2/SNF2 ATPase CHR2 remodels pri-miRNAs via Serrate to impede miRNA production. Nature.

[11] Reinhart BJ, Weinstein EG, Rhoades MW, Bartel B, Bartel DP (2002). MicroRNAs in plants. Genes Dev..

[12] Park W, Li J, Song R, Messing J, Chen X (2002). CARPEL FACTORY, a Dicer homolog, and HEN1, a novel protein, act in microRNA metabolism in *Arabidopsis thaliana*. Curr. Biol..

[13] Fukudome A, Fukuhara T (2017). Plant dicer-like proteins: double-stranded RNA-cleaving enzymes for small RNA biogenesis. J. Plant Res..

[14] Morlando M (2008). Primary microRNA transcripts are processed co-transcriptionally. Nat. Struct. Mol. Biol..

[15] Pawlicki JM, Steitz JA (2008). Primary microRNA transcript retention at sites of transcription leads to enhanced microRNA production. J. Cell Biol..

[16] Vazquez F, Gasciolli V, Crete P, Vaucheret H (2004). The nuclear dsRNA binding protein HYL1 is required for MicroRNA accumulation and plant development, but not posttranscriptional transgene silencing. Curr. Biol..

[17] Han MH, Goud S, Song L, Fedoroff N (2004). The Arabidopsis double-stranded RNA-binding protein HYL1 plays a role in microRNA-mediated gene regulation. Proc. Natl Acad. Sci. USA.

[18] Yang L, Liu ZQ, Lu F, Dong AW, Huang H (2006). SERRATE is a novel nuclear regulator in primary microRNA processing in Arabidopsis. Plant J..

[19] Lobbes D, Rallapalli G, Schmidt DD, Martin C, Clarke J (2006). SERRATE: a new player on the plant microRNA scene. EMBO Rep..

[20] Song L, Han MH, Lesicka J, Fedoroff N (2007). Arabidopsis primary microRNA processing proteins HYL1 and DCL1 define a nuclear body distinct from the Cajal body. Proc. Natl Acad. Sci. USA.

[21] Iwata Y, Takahashi M, Fedoroff NV, Hamdan SM (2013). Dissecting the interactions of SERRATE with RNA and DICER-LIKE 1 in Arabidopsis microRNA precursor processing. Nucleic Acids Res..

[22] Fang Y, Spector DL (2007). Identification of nuclear dicing bodies containing proteins for microRNA biogenesis in living Arabidopsis plants. Curr. Biol..

[23] Yu B (2005). Methylation as a crucial step in plant microRNA biogenesis. Science.

[24] Baumberger N, Baulcombe DC (2005). Arabidopsis ARGONAUTE1 is an RNA Slicer that selectively recruits rnicroRNAs and short interfering RNAs. Proc. Natl Acad. Sci. USA.

[25] Bologna NG (2018). Nucleo-cytosolic shuttling of ARGONAUTE1 prompts a revised model of the plant microRNA pathway. Mol. Cell.

[26] Qi Y, Denli AM, Hannon GJ (2005). Biochemical specialization within Arabidopsis RNA silencing pathways. Mol. Cell.

[27] Wang H (2011). Deep sequencing of small RNAs specifically associated with Arabidopsis AGO1 and AGO4 uncovers new AGO functions. Plant J..

[28] Dalmadi A, Gyula P, Balint J, Szittya G, Havelda Z (2019). AGO-unbound cytosolic pool of mature miRNAs in plant cells reveals a novel regulatory step at AGO1 loading. Nucleic Acids Res..

[29] de Felippes FF, Ott F, Weigel D (2011). Comparative analysis of non-autonomous effects of tasiRNAs and miRNAs in *Arabidopsis thaliana*. Nucleic Acids Res..

[30] Morel JB (2002). Fertile hypomorphic ARGONAUTE (ago1) mutants impaired in post-transcriptional gene silencing and virus resistance. Plant Cell.

[31] Miller JC, Chezem WR, Clay NK (2015). Ternary WD40 repeat-containing protein complexes: evolution, composition and roles in plant immunity. Front. Plant Sci..

[32] Xu C, Min J (2011). Structure and function of WD40 domain proteins. Protein Cell.

[33] Xie D (2021). Phase separation of SERRATE drives dicing body assembly and promotes miRNA processing in Arabidopsis. Nat. Cell Biol..

[34] Vaucheret H, Vazquez F, Crete P, Bartel DP (2004). The action of ARGONAUTE1 in the miRNA pathway and its regulation by the miRNA pathway are crucial for plant development. Genes Dev..

[35] Jia T (2017). The Arabidopsis MOS4-associated complex promotes MicroRNA biogenesis and precursor messenger RNA splicing. Plant Cell.

[36] Li S (2018). MAC3A and MAC3B, two core subunits of the MOS4-associated complex, positively influence miRNA biogenesis. Plant Cell.

[37] Wang S (2019). The PROTEIN PHOSPHATASE4 complex promotes transcription and processing of primary microRNAs in Arabidopsis. Plant Cell.

[38] Laubinger S (2008). Dual roles of the nuclear cap-binding complex and SERRATE in pre-mRNA splicing and microRNA processing in *Arabidopsis thaliana*. Proc. Natl Acad. Sci. USA.

[39] Zhan X (2012). Arabidopsis proline-rich protein important for development and abiotic stress tolerance is involved in microRNA biogenesis. Proc. Natl Acad. Sci. USA.

[40] Zielezinski A (2015). mirEX 2.0—an integrated environment for expression profiling of plant microRNAs. BMC Plant Biol..

[41] Kim S (2008). Two cap-binding proteins CBP20 and CBP80 are involved in processing primary MicroRNAs. Plant Cell Physiol..

[42] Ben Chaabane S (2013). STA1, an Arabidopsis pre-mRNA processing factor 6 homolog, is a new player involved in miRNA biogenesis. Nucleic Acids Res..

[43] Ren G (2012). Regulation of miRNA abundance by RNA binding protein TOUGH in Arabidopsis. Proc. Natl Acad. Sci. USA.

[44] Qiao Y, Shi J, Zhai Y, Hou Y, Ma W (2015). Phytophthora effector targets a novel component of small RNA pathway in plants to promote infection. Proc. Natl Acad. Sci. USA.

[45] Furumizu C, Tsukaya H, Komeda Y (2010). Characterization of EMU, the Arabidopsis homolog of the yeast THO complex member HPR1. RNA.

[46] Francisco-Mangilet AG (2015). THO2, a core member of the THO/TREX complex, is required for microRNA production in Arabidopsis. Plant J..

[47] Wu X (2013). A role for the RNA-binding protein MOS2 in microRNA maturation in Arabidopsis. Cell Res..

[48] Yu B (2008). The FHA domain proteins DAWDLE in Arabidopsis and SNIP1 in humans act in small RNA biogenesis. Proc. Natl Acad. Sci. USA.

[49] Zhang, S. X., Liu, Y. H. & Yu, B. PRL1, an RNA-binding protein, positively regulates the accumulation of miRNAs and siRNAs in Arabidopsis. *PloS Genetics* **10**, e1004841 (2014). 10.1371/journal.pgen.1004841 PMC4256206 25474114

[50] Iki T (2010). In vitro assembly of plant RNA-induced silencing complexes facilitated by molecular chaperone HSP90. Mol. Cell.

[51] Iwasaki S (2010). Hsc70/Hsp90 chaperone machinery mediates ATP-dependent RISC loading of small RNA duplexes. Mol. Cell.

[52] Johnston M, Geoffroy MC, Sobala A, Hay R, Hutvagner G (2010). HSP90 protein stabilizes unloaded argonaute complexes and microscopic P-bodies in human cells. Mol. Biol. Cell.

[53] Iki T, Yoshikawa M, Meshi T, Ishikawa M (2012). Cyclophilin 40 facilitates HSP90-mediated RISC assembly in plants. EMBO J..

[54] Smith MR (2009). Cyclophilin 40 is required for microRNA activity in Arabidopsis. Proc. Natl Acad. Sci. USA.

[55] Tomassi AH (2020). The intrinsically disordered protein CARP9 bridges HYL1 to AGO1 in the nucleus to promote microRNA activity. Plant Physiol..

[56] Murray MG, Thompson WF (1980). Rapid isolation of high molecular weight plant DNA. Nucleic Acids Res..

[57] Nakamura S (2010). Gateway binary vectors with the bialaphos resistance gene, bar, as a selection marker for plant transformation. Biosci. Biotechnol. Biochem..

[58] Clough SJ, Bent AF (1998). Floral dip: a simplified method for Agrobacterium-mediated transformation of *Arabidopsis thaliana*. Plant J..

[59] Liu C, Axtell MJ, Fedoroff NV (2012). The helicase and RNaseIIIa domains of Arabidopsis Dicer-Like1 modulate catalytic parameters during microRNA biogenesis. Plant Physiol..

[60] Langmead B, Trapnell C, Pop M, Salzberg SL (2009). Ultrafast and memory-efficient alignment of short DNA sequences to the human genome. Genome Biol..

[61] Nobuta K, McCormick K, Nakano M, Meyers BC (2010). Bioinformatics analysis of small RNAs in plants using next generation sequencing technologies. Methods Mol. Biol..

[62] Love MI, Huber W, Anders S (2014). Moderated estimation of fold change and dispersion for RNA-seq data with DESeq2. Genome Biol..

[63] Cai Q (2018). The disease resistance protein SNC1 represses the biogenesis of microRNAs and phased siRNAs. Nat. Commun..

[64] Jefferson RA, Kavanagh TA, Bevan MW (1987). GUS fusions: beta-glucuronidase as a sensitive and versatile gene fusion marker in higher plants. EMBO J..

[65] Li S (2020). Global co-transcriptional splicing in Arabidopsis and the correlation with splicing regulation in mature RNAs. Mol. Plant.

[66] Dobin A (2013). STAR: ultrafast universal RNA-seq aligner. Bioinformatics.

[67] Trapnell C (2013). Differential analysis of gene regulation at transcript resolution with RNA-seq. Nat. Biotechnol..

[68] Tian T (2017). agriGO v2.0: a GO analysis toolkit for the agricultural community, 2017 update. Nucleic Acids Res..

[69] Letunic I, Bork P (2021). Interactive Tree Of Life (iTOL) v5: an online tool for phylogenetic tree display and annotation. Nucleic Acids Res..

