# Supplementary material for: Arabidopsis RBV is a conserved WD40 repeat protein that promotes microRNA biogenesis and ARGONAUTE1 loading
Source: Nat Commun. 2022 Mar 8;13:1217. doi: 10.1038/s41467-022-28872-x (PMC8904849; doi:10.1038/s41467-022-28872-x)
Supplement: Supplementary file 1 — Supplementary Information [file 41467_2022_28872_MOESM1_ESM.docx]

The following Supporting Information is available for this article:**
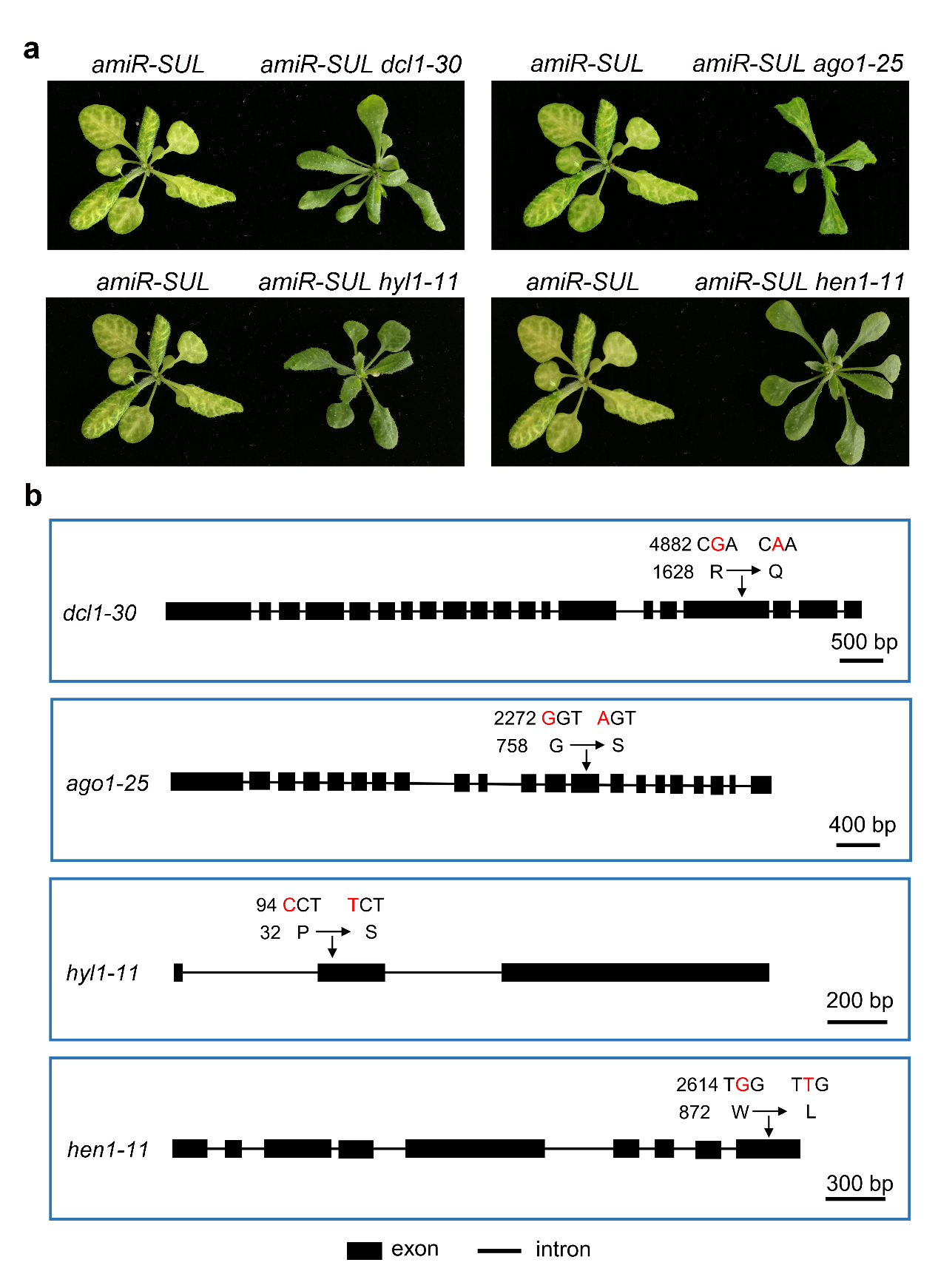
**

**Supplementary Figure 1.** Alleles of known miRNA pathway genes isolated in our genetic screen. **a** Phenotypes of 1-month-old plants of the indicated genotypes. **b** Gene structures of miRNA pathway components including *DCL1*, *AGO1*, *HYL1* and *HEN1*. New mutant alleles isolated from our screen are indicated.

**
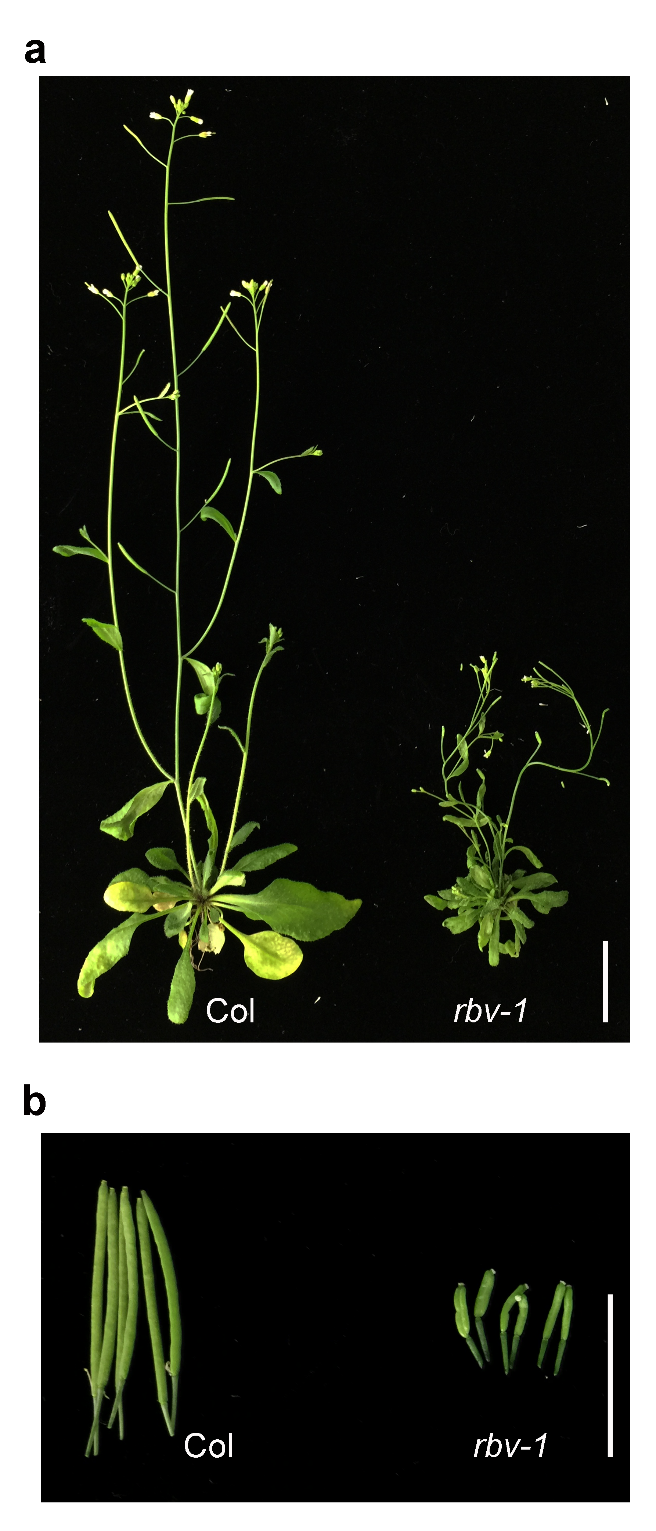
**

**Supplementary Figure 2.** Phenotypes of the *rbv-1* mutant in the Col background. **a** Phenotypes of 2-month-old Col (wild type) and *rbv1-1* plants. **b** Siliques of Col and *rbv1-1* plants. Bar = 1cm.

**
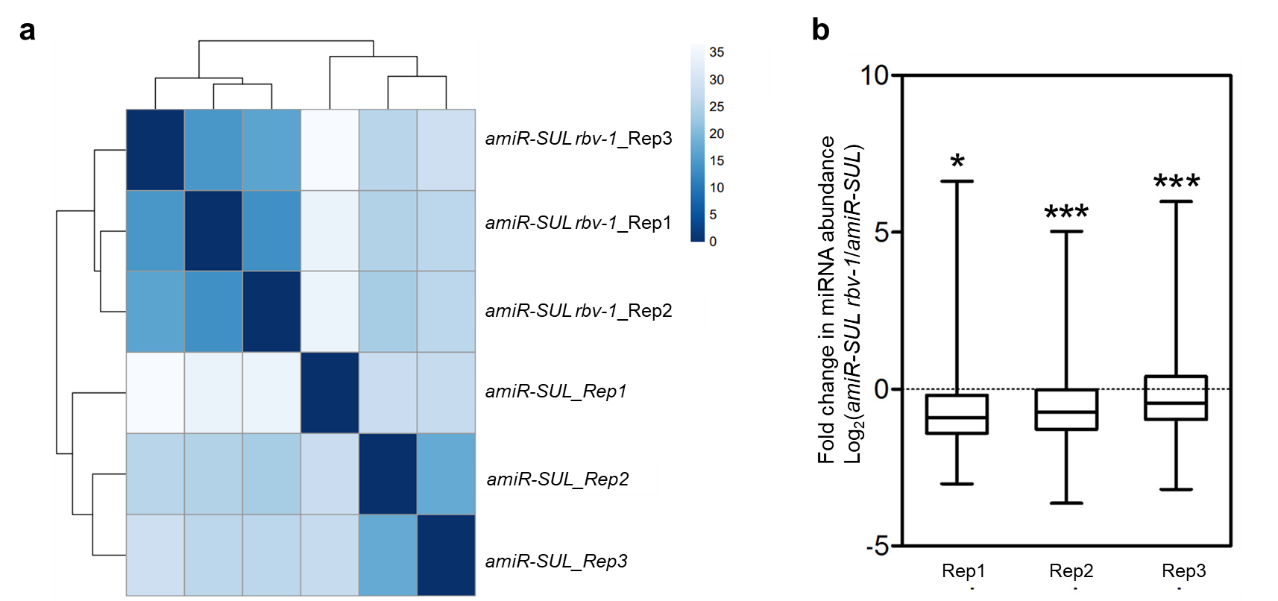
**

**Supplementary Figure 3.** Small-RNA sequencing of *amiR-SUL* and *amiR-SUL rbv-1* seedlings (Supports Fig. 1). **a** Heatmaps showing reproducibility among *amiR-SUL* and *amiR-SUL rbv-1* biological replicates. All mapped reads were used in the analysis. Sample-to-sample distances were calculated based on log2-transformed normalized read counts. The three biological replicates (Rep1, Rep2 and Rep3) for each genotype were highly reproducible. **b** Comparison of miRNA abundance between *amiR-SUL* and *amiR-SUL rbv-1* in three biological replicates (Rep1, Rep2 and Rep3). The Log_2_ratios of *amiR-SUL rbv-1*/ *amiR-SUL* were plotted. The central line of the box represents the median while two bounds represent 25% quartile and 75% quartile, respectively. The whisker represents 1.5× interquartile range of the lower or upper quartile. Three independent biological replicates were included. A global reduction in miRNA accumulation was observed in *amiRSUL rbv-1* relative to *amiR-SUL* (two tailed Student’s *t* test, * P<0.05, *** P<0.0001).


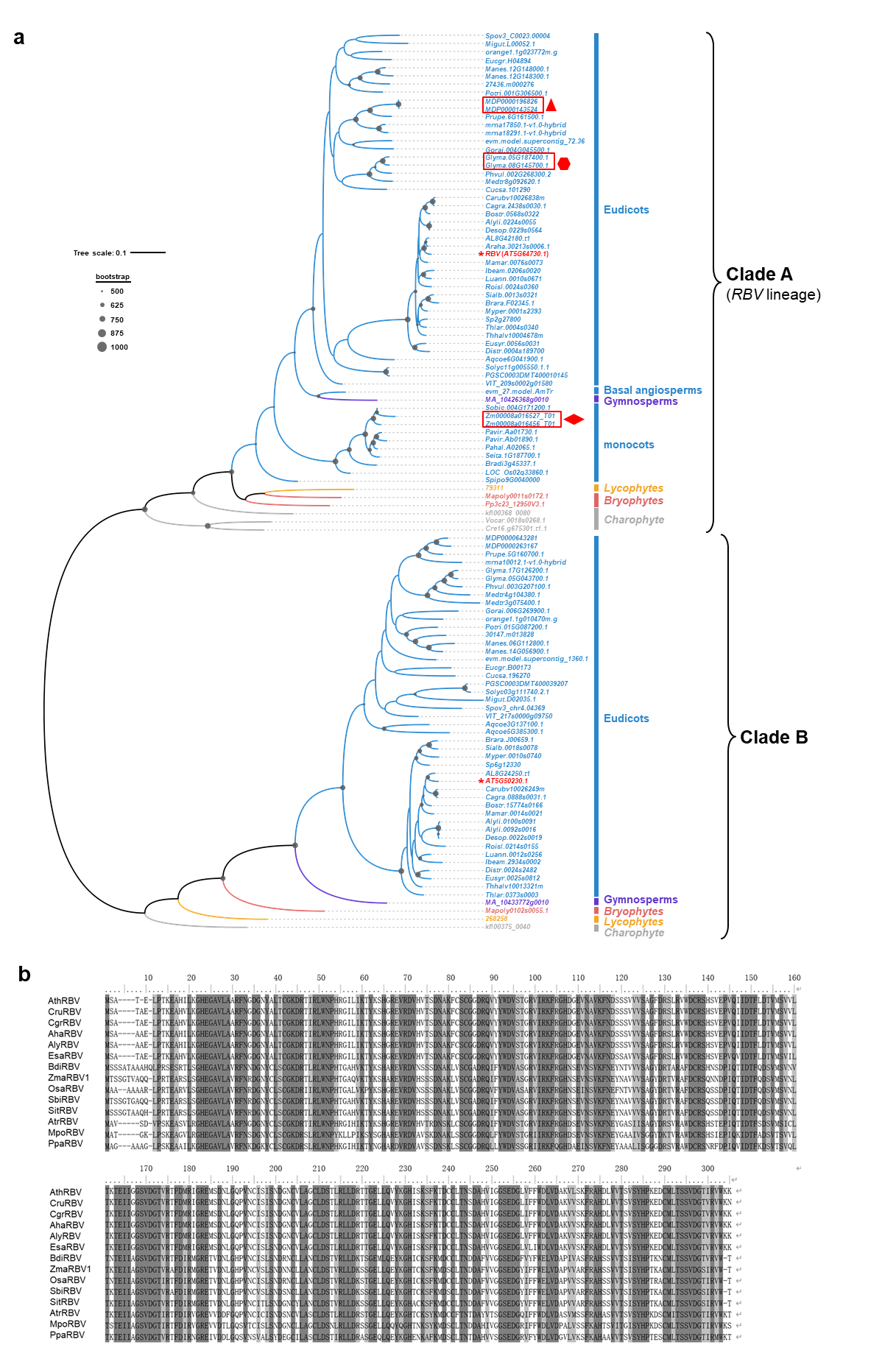


**Supplementary Figure 4.** Phylogenetic analysis of Arabidopsis *RBV* and its orthologs in different species. **a** A phylogenetic tree of Arabidopsis *RBV* orthologs in different species. The Arabidopsis *RBV* and *AT5G50230* genes are indicated by the red colored texts with asterisks. Genes in rectangles labeled with a with red triangle, hexagon and diamond are *RBVs* from apple, soybean and maize, respectively. Phytozome accessions in Supplementary Data 2 were used in this tree. **b** Amino acid sequence alignment of *Arabidopsis thaliana* RBV and its orthologs from various plants. Ath: *Arabidopsis thaliana*; Cru: *Capsella rubella*; Cgr: *Capsella grandiflora*; Aha: *Arabidopsis helleri*; Aly: *Arabidopsis lyrata*; Esa: *Eutrema salsugineum*; Bdi: *Brachypodium distachyon*; Zma: *Zea mays*; Osa: *Oryza sativa*; Sbi: *Sorghum bicolor*; Sit: *Setaria italica*; Atr: *Amborella trichopoda*; Mpo: *Marchantia polymorpha*; Ppa: *Physcomitrella patens*. The dark grey color represents amino acids that are identical among all species; the light grey color denotes amino acids that are identical in at least 50% of the species.

**
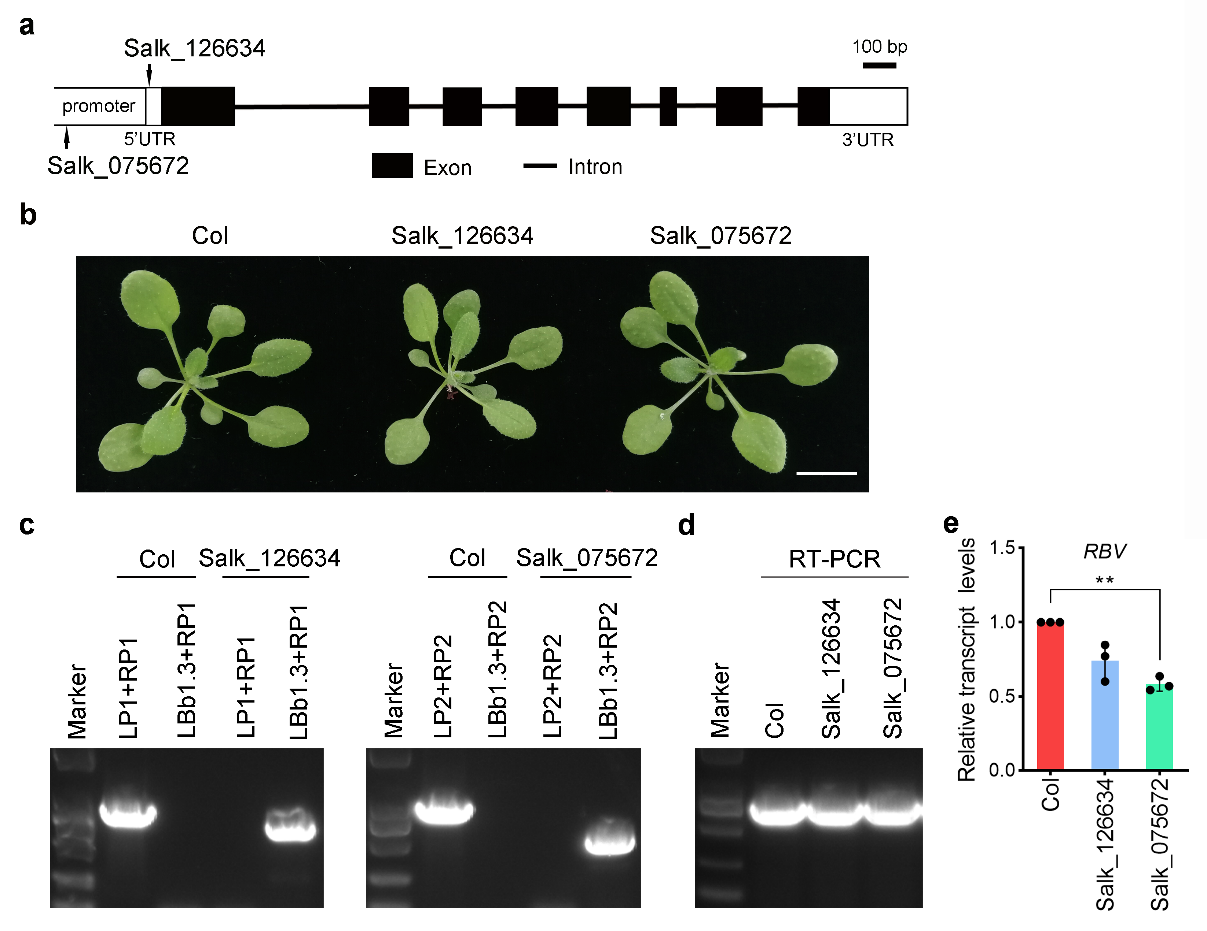
**

**Supplementary Figure 5.** Genotypes and phenotypes of two lines with T-DNA insertions near At5g64730. **a** Gene structures of *RBV* (At5g64730) (white blocks, untranslated regions; black blocks, exons; black lines, introns). The black arrows indicate the T-DNA insertion sites. Scale bar = 100bp. **b** Morphological phenotypes of 3-week-old Col, Salk_126634 and Salk_075672 plants. Scale bars = 1cm. **c** Genotyping of the two T-DNA mutants using LP (5’ genomic primer), RP (3’ genomic primer) and LBb1.3 (T-DNA left border primer) primers. **d** RT-PCR to detect *RBV* RNA from Col, Salk_126634 and Salk_075672 by amplifying the *RBV* full-length coding region. **e** RT-qPCR to determine *RBV* RNA levels from Col, Salk_126634 and Salk_075672. Three independent biological replicates were used for the calculation of standard deviation. (two-tailed Student’s *t* test, **P < 0.01)

**
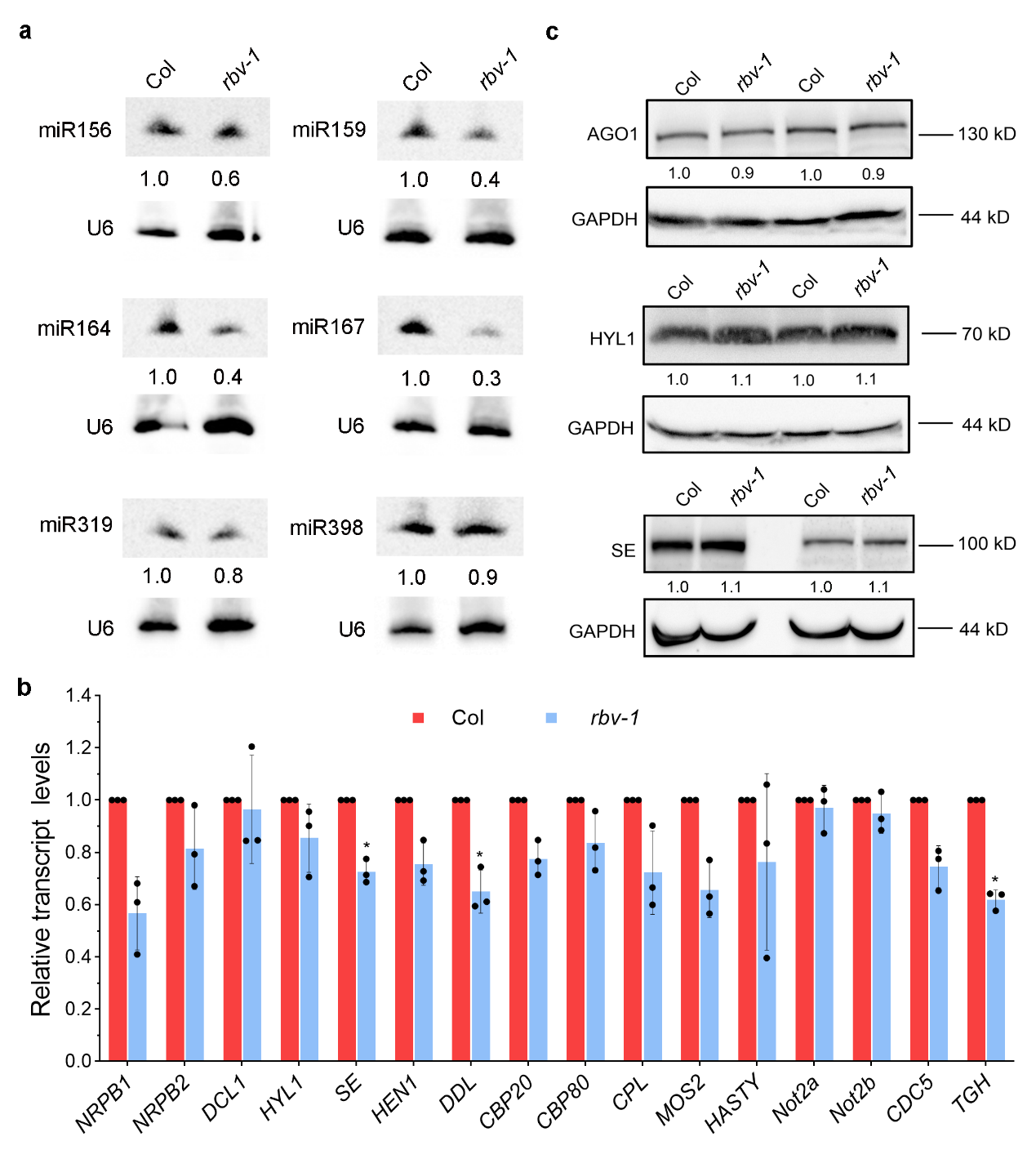
**

**Supplementary Figure 6.** The accumulation of miRNAs and the expression of known genes in the miRNA pathway in the *rbv-1* mutant. **a** Northern blots showing moderately reduced miRNA accumulation in the *rbv-1* mutant. U6 was used as an internal control. Two independent repeats gave similar results. **b** Detection of transcript levels from genes involved in miRNA biogenesis in Col and *rbv-1* by quantitative RT-PCR. Error bars represent SD of three biological replicates; Student’s *t* test was used for statistical analysis, *P<0.05. **c** Detection of AGO1, HYL1 and SE proteins in Col and *rbv-1* by western blotting. Two independent biological replicates were presented and GAPDH was used as a loading control.

**
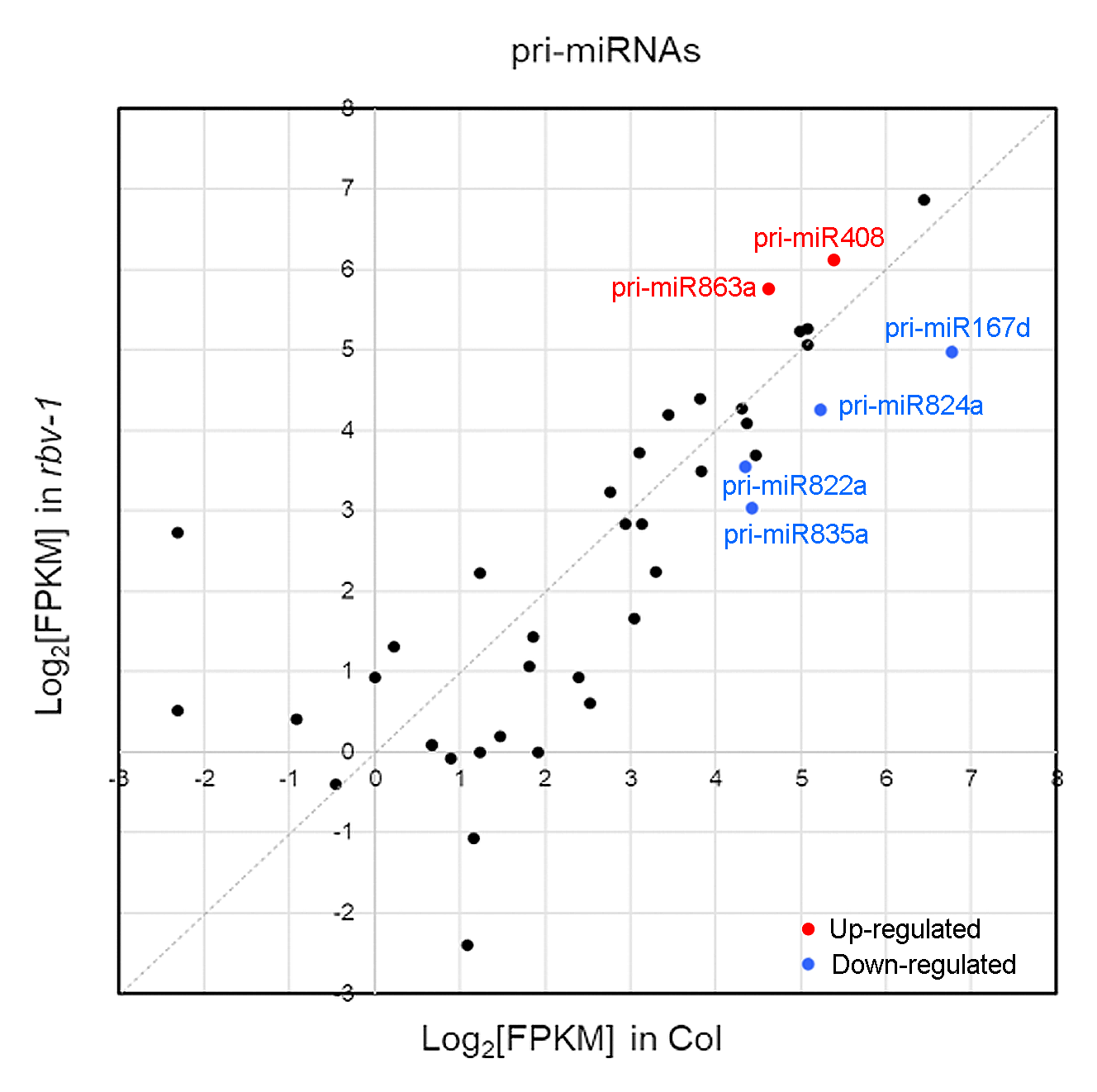
**

**Supplementary Figure 7.** RNA-Seq of WT and *rbv-1* reveals global changes in pri-miRNAs. Scatterplots showing the abundance of pri-miRNAs in *rbv-1* vs. Col. FPKM, Fragments Per Kilobase of transcript per Million mapped reads. Only the pri-miRNAs labeled in blue and red are statistically significantly different between *rbv-1* and Col.

**
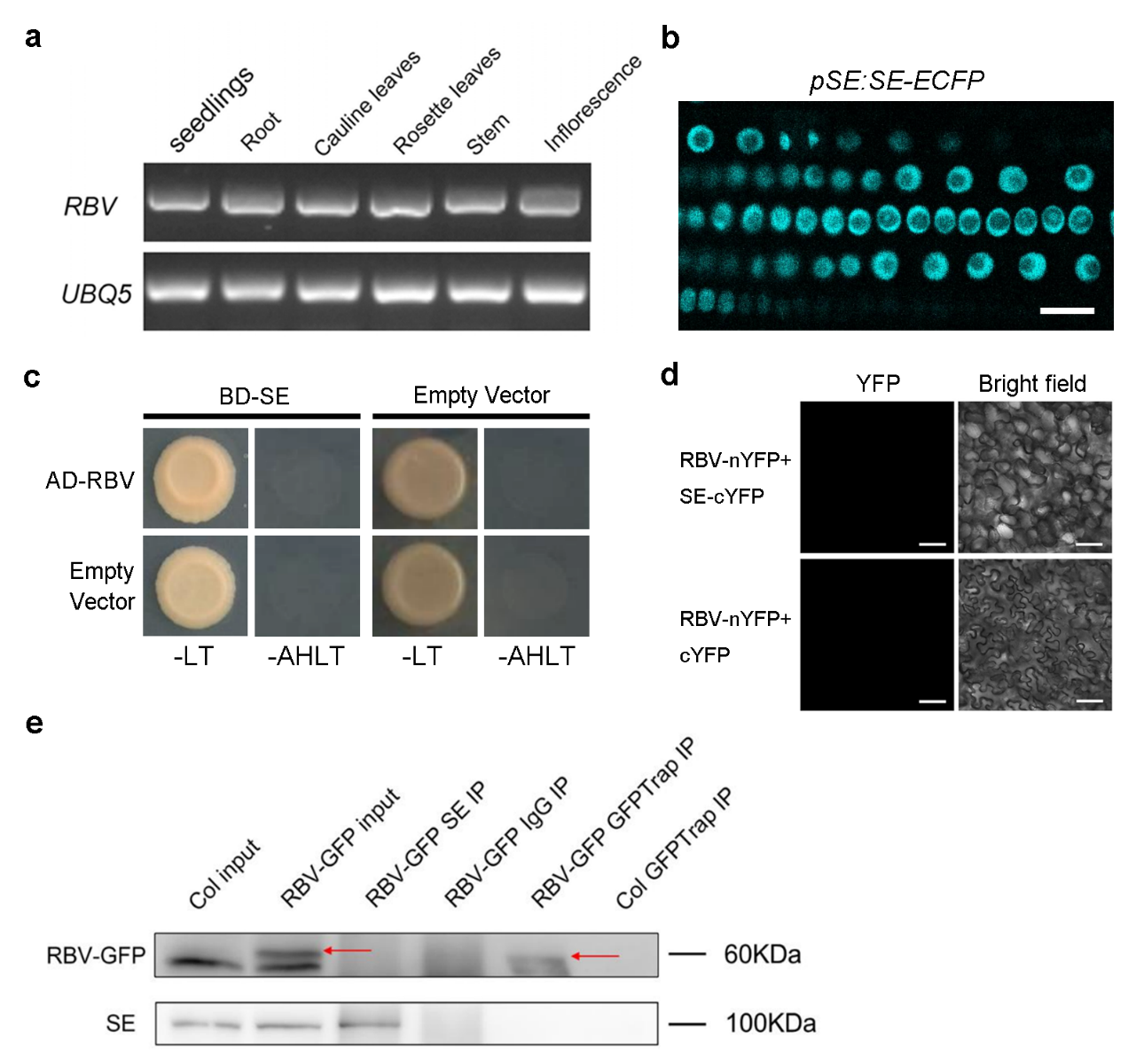
**

**Supplementary Figure 8.** ***RBV* gene expression and RBV protein information.**

**a** *RBV* is expressed in all tissues as determined by RT-PCR. *UBQ5* was used as an internal control. Three independent repeats gave similar results. **b** The subcellular localization of SE-eCFP driven by the *SE* promoter. **c** A GAL4-based yeast two-hybrid assay did not detect RBV-SE interactions. AD and BD represent the plasmids encoding the fusions to the GAL4 activation domain and the DNA-binding domain, respectively. Co-transformed yeast colonies were spotted on the selective SD medium minus Leu and Trp (-LT), then grown on SD medium minus adenine, His, Leu and Trp (-AHLT). **d** A BiFC assay did not detect RBV-SE interactions. Paired cYFP- and nYFP-fusion proteins were co-expressed in *N. benthamiana* leaves. The BiFC signals were detected by a confocal microscopy at 48 h after infiltration. Bar = 20 μm. **e** A Co-IP assay performed with *pRBV:RBV-eYFP* *rbv-1* 12-d-old seedlings did not detect RBV-SE interactions.


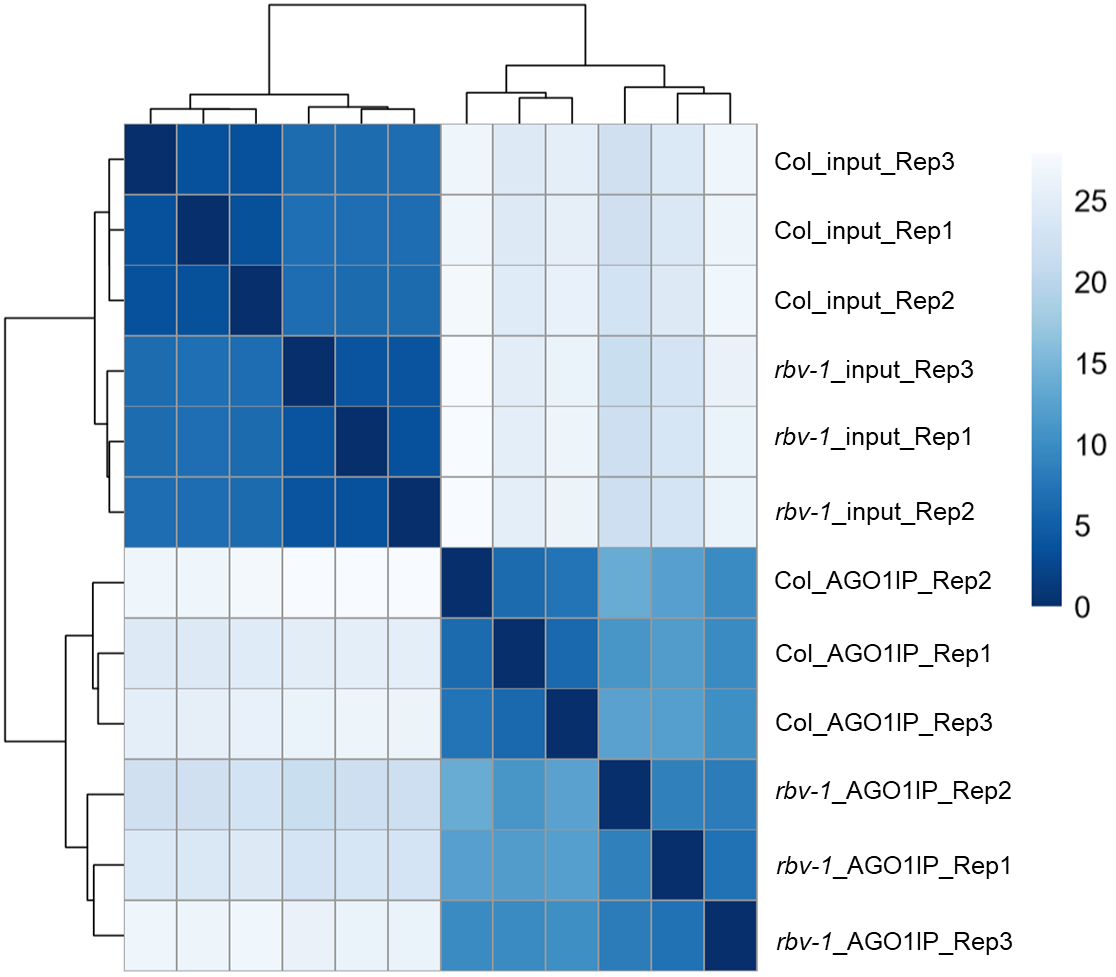


**Supplementary Figure 9.** Heatmaps showing small-RNA seq reproducibility among Col and *rbv-1* biological replicates in both input and AGO1 IP samples. Sample-to-sample distances were calculated based on log2-transformed normalized read counts. “Input” is the sample before AGO1 IP. The three biological replicates (Rep1, Rep2 and Rep3) for each treatment were highly reproducible.


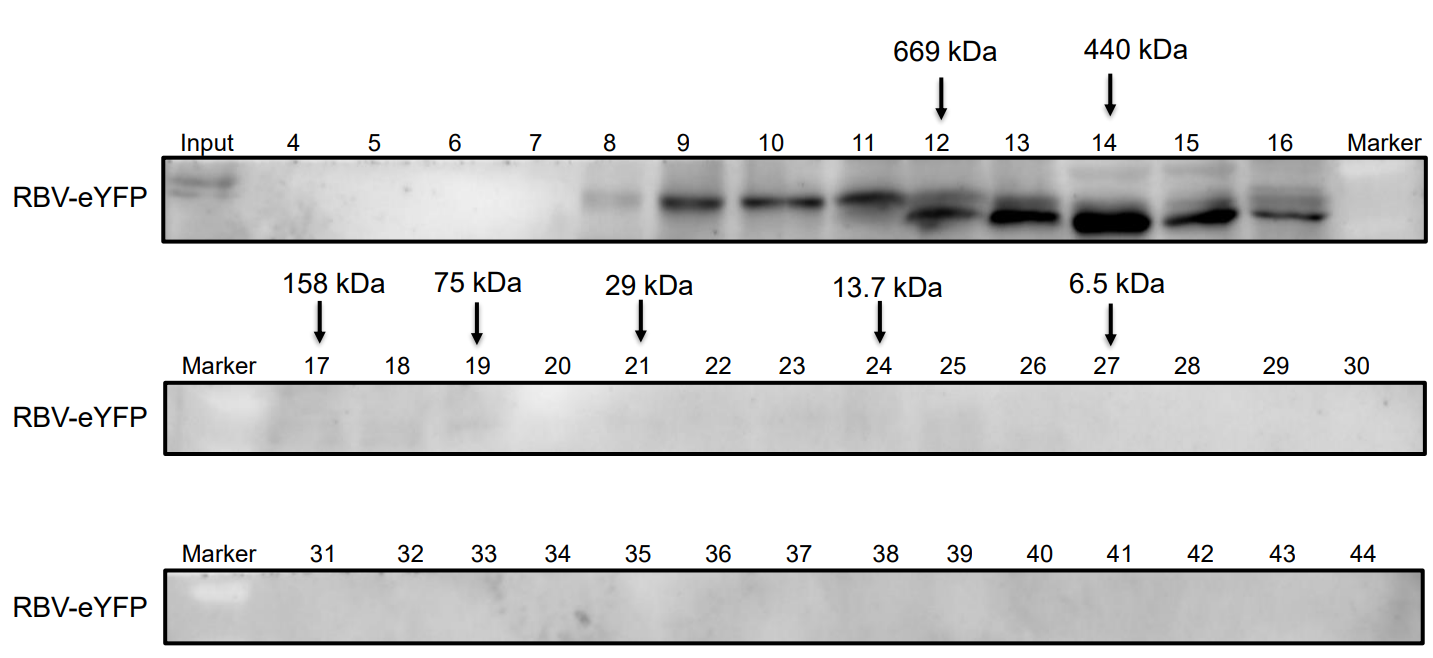


**Supplementary Figure 10.** Western blot of RBV-eYFP in gel filtration fractions. The *pRBV: RBV-eYFP rbv-1* material was used for size exclusion chromatography. The western blot with anti-GFP antibodies shows that RBV-eYFP was detected in HMW fractions 9-16. The numbers above the lanes indicate fraction numbers. The fractions where the molecular weight standards were found are indicated. Two independent repeats gave similar results.


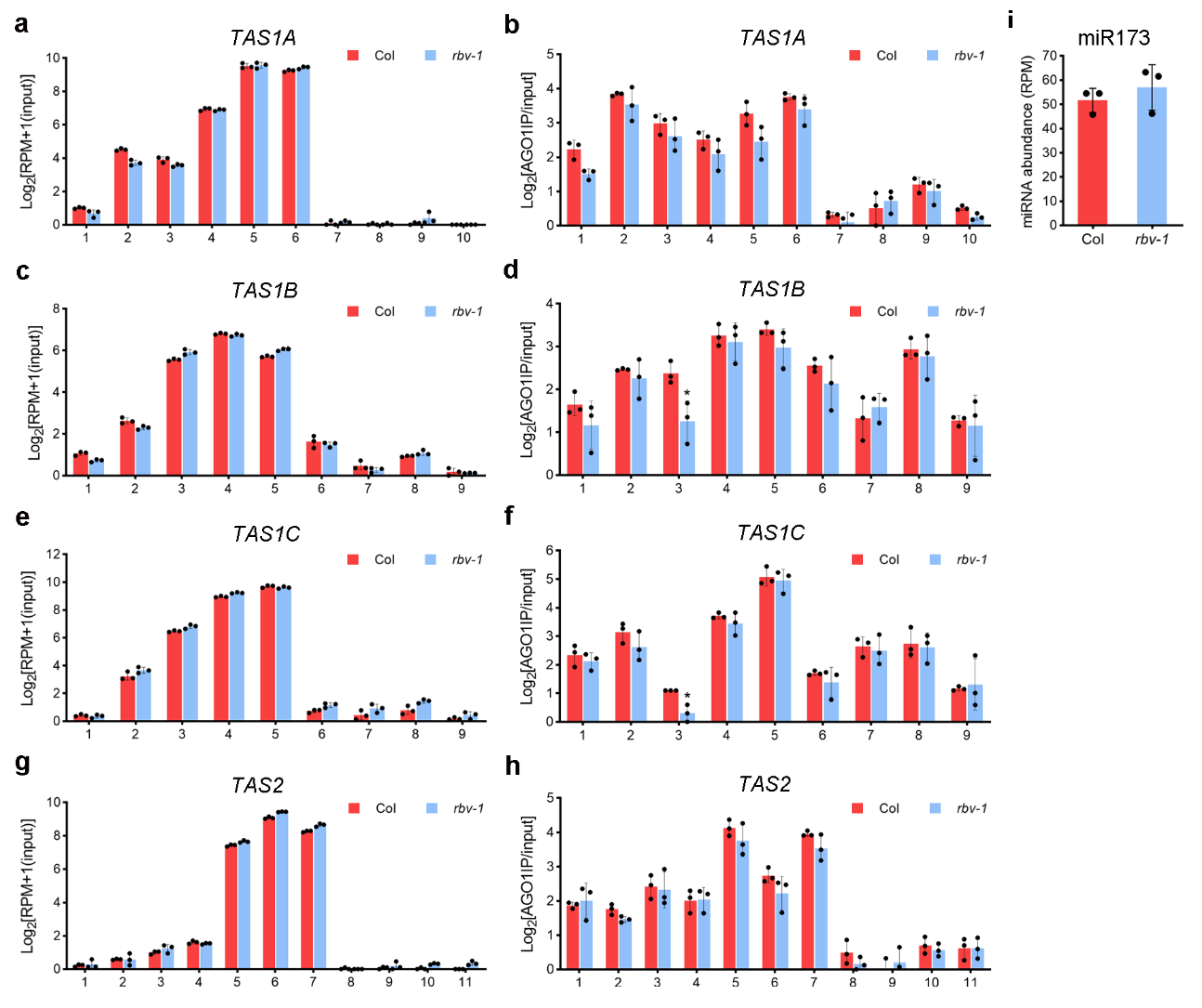


**Supplementary Figure 11.** Mutation of *RBV* did not alter the loading of tasiRNAs into AGO1. **a**,**c**,**e**,**g** Abundance of ta-siRNAs generated from *TAS1A*/*TAS1B*/*TAS1C*/*TAS2* in *rbv-1* input vs. Col input. All ta-siRNAs were normalized by total reads and Log2(RPM) value was used for plotting. Student’s *t* test was used for statistical analysis. **b**, **d**, **f**, **h** AGO1 loading efficiency of ta-siRNAs from *TAS1A*/*TAS1B*/*TAS1C*/*TAS2* loci in *rbv-1* vs. Col as determined by AGO1 IP small RNA-Seq. AGO1 loading efficiency is represented by the ratio of ta-siRNA abundance in AGO1 IP vs. input (*Student’s *t* test: *P < 0.05). In **a** to **h**, the numbers on the X-axis indicate each 100-bp window; the numbers on the Y-axis indicate the Log2 value (RPM+1) in input samples or Log2 value (AGO1 IP/Input). n=3 biologically independent experiments in each sample. Error bars indicate SD of three biological replicates; Student’s *t* test was used for statistical analysis. **i** miR173 abundance in *rbv-1* input vs. Col input. The numbers on the Y-axis indicate miR173 abundance (RPM) in each sample, n=3 biologically independent experiments. Error bars indicate SD of three biological replicates.

**
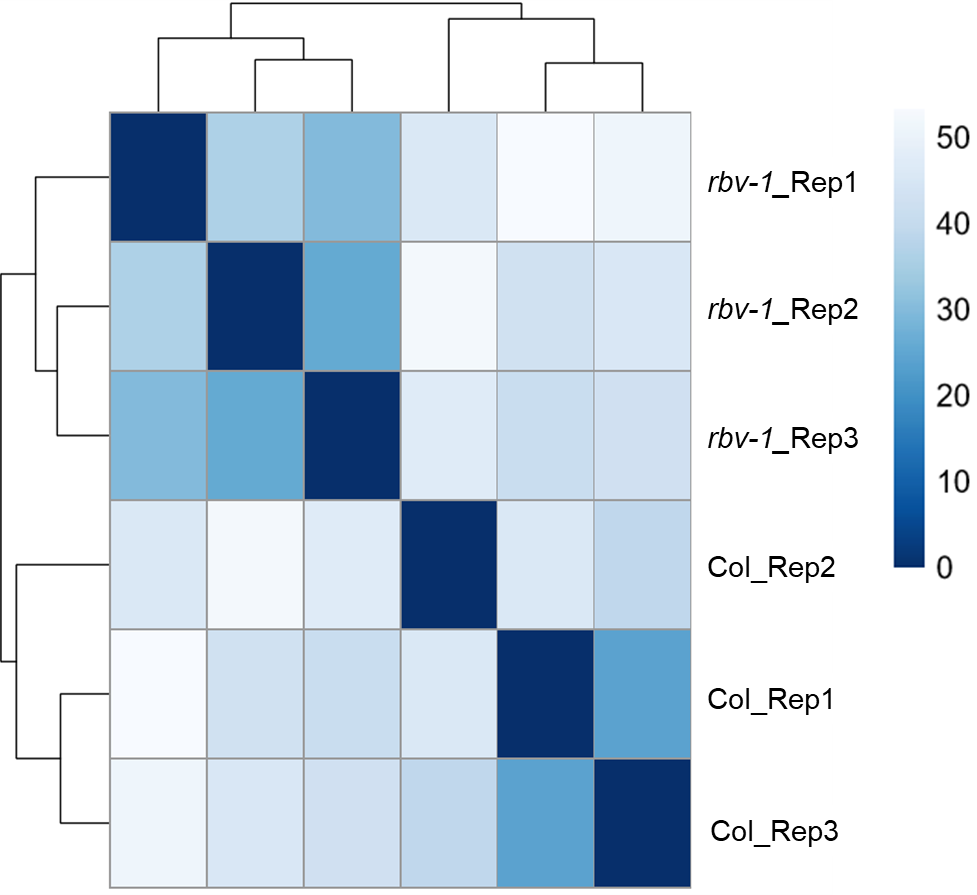
**

**Supplementary Figure 12.** Heatmaps showing the reproducibility of biological replicates of Col and *rbv-1* RNA-seq. Sample-to-sample distances were calculated based on log2-transformed normalized read counts. The three biological replicates (Rep1, Rep2 and Rep3) for each genotype were highly reproducible.

**
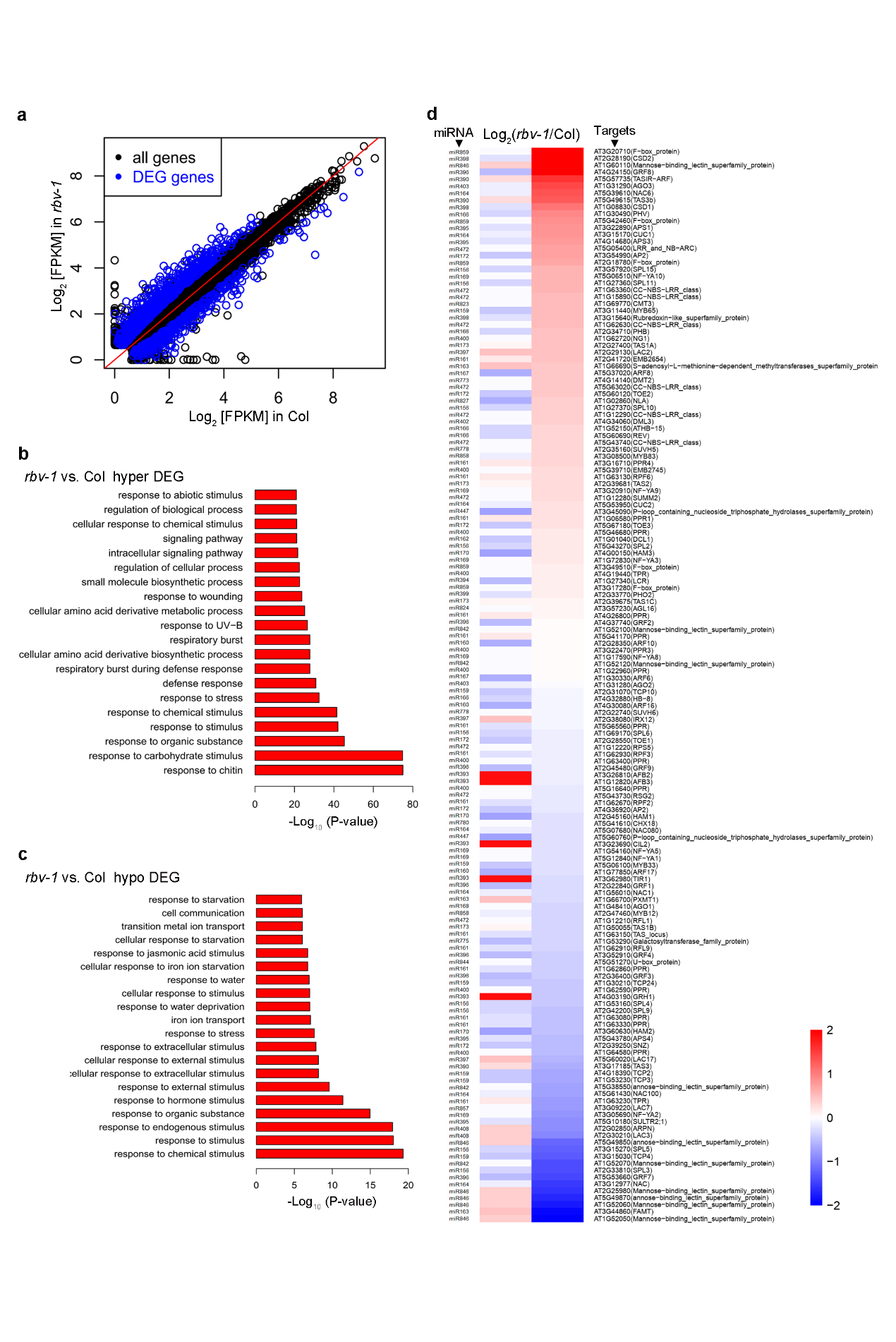
**

**Supplementary Figure 13.** RNA-seq analyses of Col and *rbv-1*. **a** A scatter plot of transcript abundance from RNA-seq of *rbv-1* and Col. Black dots, all genes; Blue dots, differentially expressed genes (DEGs) in *rbv-1* vs. Col. All genes with FPKM >1, fold-change >2 and P value <0.05 were identified as DEGs using cuffdiff. **b** The top 20 GO terms for upregulated genes (hyper-DEGs) in the *rbv-1* mutant. **c** The top 20 GO terms for downregulated genes (hypo-DEGs) in the *rbv-1* mutant. **d** Transcript levels of 155 miRNA target genes and the corresponding miRNAs in *rbv-1* vs. Col. Log2 (*rbv-1*/Col) values were used for heatmap plotting. Only transcripts with RPM >10 in either genotype (average of the three replicates) were included in the analysis. Only half of the miRNA targets showed increased expression in the *rbv-1* mutant.

**
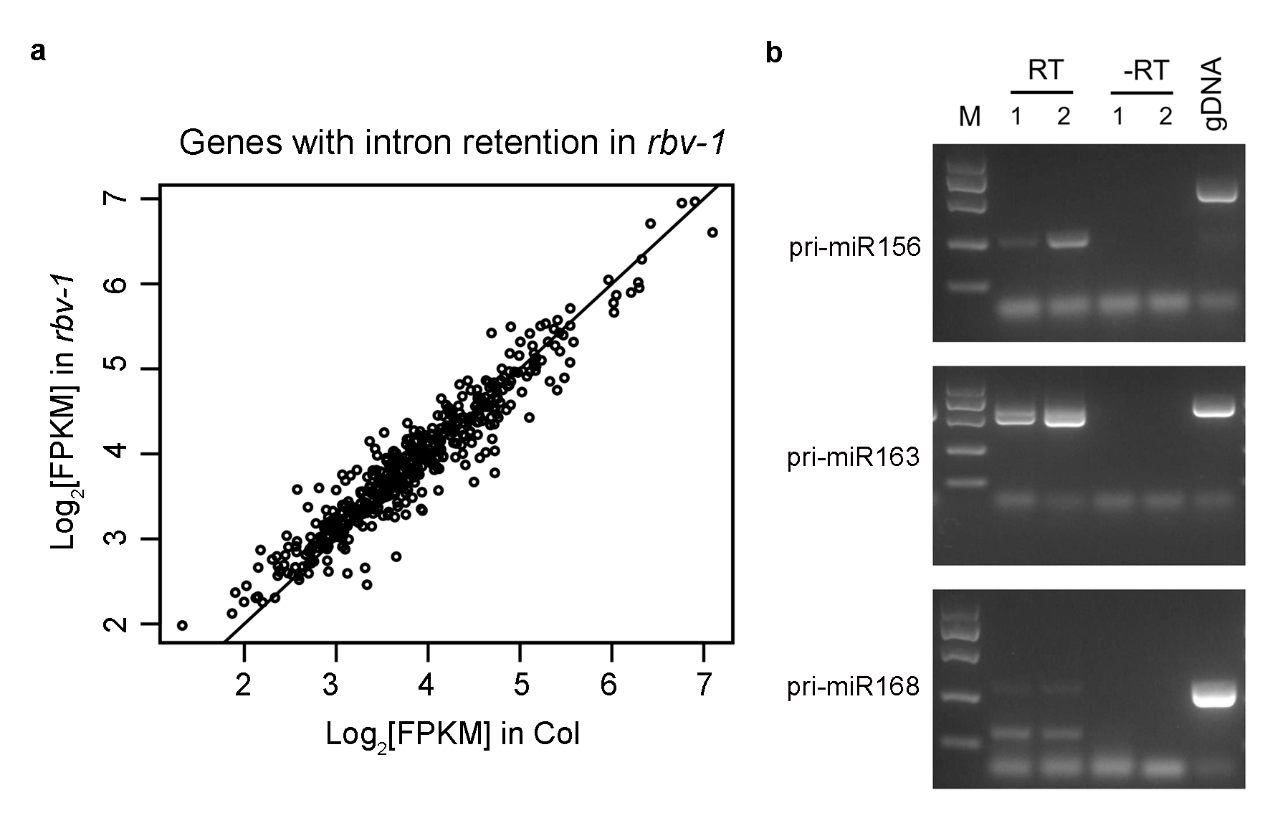
**

**Supplementary Figure 14** Analysis of gene expression and intron retention. **a** A scatter plot showing the expression of genes with intron retention in *rbv-1*. Expression levels of genes with intron retention are similar between the *rbv-1* mutant and Col. FPKM, Fragments Per Kilobase of transcript per Million mapped reads. **b** Intron-containing pri-miRNAs show no splicing defects in *rbv-1*. The splicing of some intron-containing pri-miRNAs (pri-miR156, 163 and 168) was examined by RT-PCR with intron-flanking primers. Reverse transcription was performed with an oligo dT primer. Genomic DNA (gDNA) from Col was included to indicate the expected sizes of intron-containing pri-miRNAs. The numbers represent different genotypes. 1, Col; 2, *rbv-1*; -RT, without reverse transcription. Two independent repeats gave similar results.

**Supplementary Table 1** Segregation analysis of *rbv-1* crossed to *amiR-SUL*

| F2 backcross (female×male) | Number of plants with *amiR-SUL rbv-1* phenotype | Number of total F2 plants | Plants with *amiR-SUL rbv-1* phenotype/total (expected) | χ^2^ (*P* value) for 3:1 |
| --- | --- | --- | --- | --- |
| *amiR-SUL* × *amiR-SUL rbv-1* | 141 | 614 | 23%（25%） | 1.357(0.244) |

**Supplementary Table 2.** Sequences of oligonucleotides used in this study

| **Oligonucleotides** | **Sequence (5'-3')** |
| --- | --- |
| **Primers for cloning or genotyping** | |
| RBV_promoter_F_KpnI | CAGGGTACCAGATGTTTAATTTAACCGGG |
| RBV_promoter_R_SacI | AATGAGCTCCTCTCTCGCCTGAAACTTA |
| RBV_genome_F_KpnI | CAGGGTACCAGATGTTTAATTTAACCGGG |
| RBV_genome_R_SalI | CGGGTCGACTTTTTTCCAGACACGAAT |
| RBV_genotyping_F | AGCGGCGAGGTTTAACGGCCATG |
| RBV_genotyping_R | GGACTTCGCGGCCATGAGAT |
| RBV_CDS_F_BamHI | ACGGGATCCATGAGCGCGACGGAGCTG |
| RBV_CDS_R_SalI | AAGGTCGACTTTTTTCCAGACACGAAT |
| LP1 | ATGGCTCCACAACAATCTTTG |
| RP1 | CGATCGTTTATAGCTGCAAGC |
| LBb1.3 | ATTTTGCCGATTTCGGAAC |
| LP2 | CGATCGTTTATAGCTGCAAGC |
| RP2 | ATGGCTCCACAACAATCTTTG |
| **Probes for small RNA northern blots** | |
| amiR-SUL_probe_Biotin | AGGGATTTCCGTGACACTTAA |
| miR156_probe_Biotin | GTGCTCACTCTCTTCTGTCA |
| miR159_probe_Biotin | TAGAGCTCCCTTCAATCCAAA |
| miR163_probe_Biotin | ATCGAAGTTCCAAGTCCTCTTCAA |
| miR164_probe_Biotin | TGCACGTGCCCTGCTTCTCCA |
| miR167_probe_Biotin | TAGATCATGCTGGCAGCTTCA |
| miR166_probe_Biotin | GGGGAATGAAGCCTGGTCCGA |
| miR319_probe_Biotin | GGGAGCTCCCTTCAGTCCAA |
| miR390_probe_Biotin | GGCGCTATCCCTCCTGAGCTT |
| miR398_probe_Biotin | AAGGGGTGACCTGAGAACACA |
| miR845_probe_Biotin | CATCAATTGGTATCAGAGCCG |
| U6_probe_Biotin | AGGGGCCATGCTAATCTTCTCTG |
| tRNA^met^ | TCGAACTCTCGACCTCAGGAT |
| **Primers for quantitative RT-PCR detection of pri-miRNAs** | |
| qPCR-pri-miR156a-F | GAAAGAGTTGGGACAAGAGAAACG |
| qPCR-pri-miR156a-R | AGAGAACGAAGACAGGCCAAAGA |
| qPCR-pri-miR159a-F | TCTTTACAGTTTGCTTATGTCAGATCCA |
| qPCR-pri-miR159a-R | ACCCTGCTCAACTCATGTTTGAA |
| qPCR-pri-miR159b-F | TGCTTGGATCTCTAATGCTGTTCA |
| qPCR-pri-miR159b-R | TCACCCTGCTAAACCCTCCA |
| qPCR-pri-miR166a-F | AGATATATATTCAGAAACCCTAG |
| qPCR-pri-miR166a-R | GGTTCATTCACTGGATCTGAAAC |
| qPCR-pri-miR167a-F | TGATCTGCTACGGTGAAGTCTATGG |
| qPCR-pri-miR167a-R | GAAACTGCGAACATGATCTAATCGA |
| qPCR-pri-miR167b-F | TCTTTGGTTAAGAGATGAATGTGGAA |
| qPCR-pri-miR167b-R | ATTTTTCTTTCAATCGGCATGTG |
| qPCR-pri-miR319b-F | GTGCTTGTATGTATGCGTATATATAGT |
| qPCR-pri-miR319b-R | CTCAATTTGTCTCTCGCATCATTC |
| **Primers for quantitative RT-PCR detection of miRNA targets** | |
| qSPL5_F | GGAGACGAAGACAAAAGGAAGA |
| qSPL5_R | CCTTTGCATGTACTTCACATAC |
| qPHB_F | TGACTGTTGCTGCTTTGAGACAT |
| qPHB_R | CCATTGGTGACCATCCATCAT |
| qPHV_F | ATCGTTGATCATGTGGACTTGGA |
| qPHV_R | CTATACTGGACTTCTCCACTAGT |
| qREV_F | ACTTTGGCAGAGTTCCTATCCAA |
| qREV_R | CGATCTTTGAGGATCTCTGCAA |
| qMYB33_F | AGTTGTTGTATCCTGGGTGTAGCA |
| qMYB33_R | CCGTTGGTGGTGGTGGAGAC |
| qARF8_F | AGATGTTTGCTATCGAAGGGTTGTTG |
| qARF8_R | CCATGGGTCATCACCAAGGAGAAG |
| qAGO1_F | GCCTTCATAGAGGCAAACCCT |
| qAGO1_R | GTACTTCCGGCGCATGTTTC |
| qSPL10_F | TGAGACAAAGCCTACACAGATGGA |
| qSPL10_R | GATGATGCAACCCGACTTTTTTATG |
| qSPL3_F | CTGGACACAACGAGAGAAG |
| qSPL3_R | TGGAGAAACAGACAGAGACA |
| qMYB65_F | AATCTGACTCCTCTACCTGCG |
| qMYB65_R | ATTCGTCTCATATGATGATGG |
| qCSD2_F | ACAGGACCACATTTCAACCCTAA |
| qCSD2_R | CATCGGCATTGGCATTTATG |
| qCUC2_F | GCACCAACACAACCGTCACAG |
| qCUC2_R | GAATGAGTTAACGTCTAAGCCCAAGG |
| qPCR-UBQ5_F | GGTGCTAAGAAGAGGAAGAA |
| qPCR-UBQ5_R | CTCCTTCTTTCTGGTAAACGT |
| **Primers for quantitative RT-PCR detection of genes in miRNA biogenesis** | |
| qNRPB1_F | TGCTGCGGCTTATGCTGAGAC |
| qNRPB1_R | GTGCCTGACACTGGTGAACGA |
| qNRPB2_F | ACGTGGTGCAACTGTTGGTGTA |
| qNRPB2_R | AAGCAGCAGCCGGTGTATGAT |
| qDCL1_F | TGGAATGACTGCTTCGCCTGTT |
| qDCL1_R | ACTTGCTTGTGCCGCTTCTTCA |
| qHYL1_F | GCTCCCAACGCCTGTTTATG |
| qHYL1_R | CGACCTTCTGGCACTGATACA |
| qSE_F | AACCAGCCACGCAGCAATCT |
| qSE_R | CGTCCACCTCTATCATCCCTCA |
| qHEN1_F | GTTGACATCGGCACTTGCTTA |
| qHEN1_R | TGGGTTTCTGGTGTAGACCG |
| qDDL1_F | AAGAGGCTCTGGCAGCGAAGA |
| qDDL1_R | GGTGAAGGCAGAGTGGCTCATT |
| qCBP20_F | GGCAGCTCGTGGATTACGGTAC |
| qCBP20_R | CACCATCGTCATCGGAGTCACC |
| qCBP80_F | CAGCCGAATTGAGCAGGAAGGT |
| qCBP80_R | GCCATATCGCTCCAGGACAGTG |
| qCPL_F | TGACTTGCTTGCTCGCATCGT |
| qCPL_R | CCTCGGCTGATCCTTCTCATCC |
| qMOS2_F | TGGTGGTGATGCGGTTGAAGAG |
| qMOS2_R | CCATCAACGCAGCACCGAAAC |
| qHASTY_F | GAACACGACCTCTGCGACAACT |
| qHASTY_R | AGATTCACTTGCCTCGGCTTGT |
| qNOT2a_F | CCAACAACAAAATGGAAGTATTTCGAA |
| qNOT2a_R | CCAACCATATTCCCCATAGA |
| qNOT2b_F | CTAAGGCCAAGACCCTGTAG |
| qNOT2b_R | ATCCAAAATGAAGACTTTCC |
| qCDC5_F | ACTCCTGGTGGTGCTGGTCTT |
| qCDC5_R | GCTTCCTCTCGTCTCTGCCTCT |
| qTGH_F | GATGGCGGCGTGGTCATTCAAT |
| qTGH_R | AGCGAGTCGGGCGTTTCCTT |
| qPCR-UBQ5_F | GGTGCTAAGAAGAGGAAGAA |
| qPCR-UBQ5_R | CTCCTTCTTTCTGGTAAACGT |
| qPCR-RBV_F | TTTTAGCGGCGAGGTTTA |
| qPCR-RBV_R | GGCATTGTCTGAAGTAAC |
| **Primers for quantitative RT-PCR detection of other genes** | |
| qPCR-SUL_F | GATCCAAAGATTGGTGGTGTTATG |
| qPCR-SUL_R | AACTTGCTCTCCTTTCTCAACTCT |
| qPCR-UBQ5_F | GGTGCTAAGAAGAGGAAGAA |
| qPCR-UBQ5_R | CTCCTTCTTTCTGGTAAACGT |
| qPCR-GUS_F | TCAGGAAGTGATGGAGCA |
| qPCR-GUS_R | AGAGCATTACGCTGCGAT |
| **Primers for quantitative RT-PCR in CHIP** | |
| qPCR-MIR166a-Chip_F | TGGCTCTCTCCACTACTCAA |
| qPCR-MIR166a-Chip_R | GACAACAGTCCCCTCAAAA |
| qPCR-MIR167a-Chip_F | CGACCCTTAAACTCTCCATAA |
| qPCR-MIR167a-Chip_R | ACTTCACCGTAGCAGATCAA |
| qPCR-MIR171a-Chip_F | TGCTTTGGTAGTAGATGAGGTT |
| qPCR-MIR171a-Chip_R | CGTGTGTGGTCAGGTAAGAT |
| qPCR-Pol II-C1-F | AGTTCAATGGAGAGATGTCGAAATATG |
| qPCR-Pol II-C1-R | AAGAGGAAAAGAAAGAGATGGAGAGA |
