# Supplementary material for: Arabidopsis RBV is a conserved WD40 repeat protein that promotes microRNA biogenesis and ARGONAUTE1 loading
Source: Nat Commun. 2022 Mar 8;13:1217. doi: 10.1038/s41467-022-28872-x (PMC8904849; doi:10.1038/s41467-022-28872-x)
Supplement: Supplementary file 2 — Description of Additional Supplementary Files [file 41467_2022_28872_MOESM2_ESM.pdf]

Description of Additional Supplementary Files

File name: Supplementary Data 1

Description: miRNAs from small RNA sequencing of *amiR-SUL* and *amiR-SUL rbv-1*

File name: Supplementary Data 2

Description: IDs of genes used in the phylogenetic analysis

File name: Supplementary Data 3

Description: Small RNA reads in *rbv-1* and Col input samples

File name: Supplementary Data 4

Description: Small RNA reads in *rbv-1* and Col AGO1 IP samples

File name: Supplementary Data 5

Description: The 632 hyper-DEGs in *rbv-1* vs. Col

File name: Supplementary Data 6

Description: The 363 hypo-DEGs in *rbv-1* vs. Col

File name: Supplementary Data 7

Description: Expression levels of 157 miRNA targets in *rbv-1* vs. Col

File name: Supplementary Data 8

Description: List of genes with intron retention in the *rbv-1* mutant
