# Supplementary material for: Arabidopsis RBV is a conserved WD40 repeat protein that promotes microRNA biogenesis and ARGONAUTE1 loading
Source: Nat Commun. 2022 Mar 8;13:1217. doi: 10.1038/s41467-022-28872-x (PMC8904849; doi:10.1038/s41467-022-28872-x)
Supplement: Supplementary file 11 — Reporting Summary [file 41467_2022_28872_MOESM11_ESM.pdf]

nature portfolio

Corresponding author(s): Xuemei Chen

Last updated by author(s): Dec 6, 2021

Reporting Summary

Nature Portfolio wishes to improve the reproducibility of the work that we publish. This form provides structure for consistency and transparency in reporting. For further information on Nature Portfolio policies, see our Editorial Policies and the Editorial Policy Checklist.

Statistics

For all statistical analyses, confirm that the following items are present in the figure legend, table legend, main text, or Methods section.

n/a	Confirmed	
☐	☒	The exact sample size (*n*) for each experimental group/condition, given as a discrete number and unit of measurement
☐	☒	A statement on whether measurements were taken from distinct samples or whether the same sample was measured repeatedly
☐	☒	The statistical test(s) used AND whether they are one- or two-sided<br>*Only common tests should be described solely by name; describe more complex techniques in the Methods section.*
☒	☐	A description of all covariates tested
☐	☒	A description of any assumptions or corrections, such as tests of normality and adjustment for multiple comparisons
☐	☒	A full description of the statistical parameters including central tendency (e.g. means) or other basic estimates (e.g. regression coefficient) AND variation (e.g. standard deviation) or associated estimates of uncertainty (e.g. confidence intervals)
☐	☒	For null hypothesis testing, the test statistic (e.g. *F*, *t*, *r*) with confidence intervals, effect sizes, degrees of freedom and *P* value noted<br>*Give P values as exact values whenever suitable.*
☒	☐	For Bayesian analysis, information on the choice of priors and Markov chain Monte Carlo settings
☒	☐	For hierarchical and complex designs, identification of the appropriate level for tests and full reporting of outcomes
☒	☐	Estimates of effect sizes (e.g. Cohen's *d*, Pearson's *r*), indicating how they were calculated

Our web collection on statistics for biologists contains articles on many of the points above.

Software and code

Policy information about availability of computer code

Data collection	Small RNA libraries were prepared with NEBNext Multiplex Small RNA Library Prep Set for Illumina (New England Biolabs, E7300), and then sequenced using an Illumina HiSeq2500 platform at BerryGenomics, China.<br>Total RNAs extracted from 14-day-old seedlings were sent for library construction at Novogene, China and libraries were sequenced on an Illumina Hiseq 4000 platform to generate paired-end reads of 150bp in length.
Data analysis	For small RNAs in amiR-SUL background, data analysis was performed with the pRNASeqTools pipeline (https://github.com/grubbybio/pRNASeqTools).<br>For AGO1 IP sRNA data, the data was analyzed using the script developed by Dr. Gao Lei (https://github.com/liangchao1979/Plant-sRNA-analysis)<br>The RNA-Seq data was analyzed according the reference: Trapnell C, Hendrickson DG, Sauvageau M, Goff L, Rinn JL, Pachter L. Differential analysis of gene regulation at transcript resolution with RNA-seq. Nat Biotechnol 31, 46-+ (2013).<br>The analysis of splicing defects was carried out using Araport 11 intron annotation and a previously developed pipeline known as SQUID (https://github.com/sfli001/SQUID)

For manuscripts utilizing custom algorithms or software that are central to the research but not yet described in published literature, software must be made available to editors and reviewers. We strongly encourage code deposition in a community repository (e.g. GitHub). See the Nature Portfolio guidelines for submitting code & software for further information.

Data

Policy information about availability of data

All manuscripts must include a data availability statement. This statement should provide the following information, where applicable:

- Accession codes, unique identifiers, or web links for publicly available datasets
- A description of any restrictions on data availability
- For clinical datasets or third party data, please ensure that the statement adheres to our policy

All raw and processed data were deposited at NCBI GEO (http://www.ncbi.nlm.nih.gov/geo/) with the accession number GSE152911 [https://www.ncbi.nlm.nih.gov/geo/query/acc.cgi?acc=GSE152911]. Source data are provided with this paper.

Field-specific reporting

Please select the one below that is the best fit for your research. If you are not sure, read the appropriate sections before making your selection.

☒ Life sciences ☐ Behavioural & social sciences ☐ Ecological, evolutionary & environmental sciences

For a reference copy of the document with all sections, see nature.com/documents/nr-reporting-summary-flat.pdf

Life sciences study design

All studies must disclose on these points even when the disclosure is negative.

Sample size	15-day-old Arabidopsis seedlings, as the whole seedlings are suitable for a set of experiments.
Data exclusions	No data were excluded from the analyses.
Replication	Three biological replicates were used in this study.
Randomization	This is not relevant to this study.
Blinding	This is not relevant to this study.

Reporting for specific materials, systems and methods

We require information from authors about some types of materials, experimental systems and methods used in many studies. Here, indicate whether each material, system or method listed is relevant to your study. If you are not sure if a list item applies to your research, read the appropriate section before selecting a response.

Materials & experimental systems

n/a	Involved in the study	
☐	☒	Antibodies
☒	☐	Eukaryotic cell lines
☒	☐	Palaeontology and archaeology
☒	☐	Animals and other organisms
☒	☐	Human research participants
☒	☐	Clinical data
☒	☐	Dual use research of concern

Methods

n/a	Involved in the study	
☒	☐	ChIP-seq
☒	☐	Flow cytometry
☒	☐	MRI-based neuroimaging

Antibodies

Antibodies used	Antibodies used included RPB1 (Abcam, ab817, dilution, 1:200), anti-GAPDH (Santa Cruz Biotechnology, sc-365062, dilution, 1:1000), anti-SUL (dilution, 1:1000) 37, anti-HYL1 (Agrisera, AS06136, dilution, 1:2000), anti-AGO1 (Agrisera, AS09527, dilution, 1:2000), anti-SE (Agrisera, AS09532A, dilution, 1:1000), anti- H3 (Agrisera, AS10710, dilution 1:3000) and anti-GFP (Abcam, ab290, dilution, 1:3000).<br>The secondary antibodies were horseradish peroxidase-conjugated, goat-anti-rabbit IgG (Bio-Rad, cat.172-1019) (dilution, 1:2000) or goat-anti-mouse IgG (Bio-Rad, cat.170-6516, dilution, 1:2000).
Validation	We have validated these antibodies in this study.
